# Assessment of Heterosexual-Identified Men Who Have Sex With Men and Men of Diverse Sexual Identities: Protocol for an International, Multilingual, Online, Comparative Sexuality Study

**DOI:** 10.2196/66897

**Published:** 2025-04-30

**Authors:** Andrew D Eaton, Travis R Scheadler, Megan Rowe, Salem Rao, Sandra Kwan, Oliver W J Beer, Paul A Shuper, Tyrone J Curtis, Adam Busch, Daniel Vandervoort, Lauren B McInroy

**Affiliations:** 1 Faculty of Social Work Saskatoon Campus University of Regina Saskatoon, SK Canada; 2 Factor-Inwentash Faculty of Social Work University of Toronto Toronto, ON Canada; 3 College of Social Work The Ohio State University Columbus, OH United States; 4 Faculty of Social Work Dalhousie University Halifax, NS Canada; 5 Social Work University of Plymouth Plymouth United Kingdom; 6 Centre of Health Technology University of Plymouth Plymouth United Kingdom; 7 Institute for Mental Health Policy Research Centre for Addiction and Mental Health Toronto, ON Canada; 8 Campbell Family Mental Health Research Institute Centre for Addiction and Mental Health Toronto, ON Canada; 9 Department of Psychiatry and Dalla Lana School of Public Health University of Toronto Toronto, ON Canada; 10 School of Public Health and Social Policy Faculty of Human and Social Development University of Victoria Victoria, BC Canada; 11 HQ Toronto Toronto, ON Canada

**Keywords:** heterosexual-identified men who have sex with men, sexuality, survey, interviews, structural equation modeling, interpretive phenomenology

## Abstract

**Background:**

Sexuality is multidimensional and complex, and involves identity development, attraction, and behavior. Heterosexual-identified men who have sex with men (H-MSM) experience sexual identity and behavior discordance, yet it is unknown how H-MSM compare to concordant heterosexual men and gay, bisexual, and queer (GBQ+) men in terms of sexuality constructs.

**Objective:**

This study aims to survey adult cisgender men in Canada, the United States, and the United Kingdom to gain greater insights into how demographics, identity development, attraction, behavior, technology use, relationship negotiation and communication skills, and pre-exposure prophylaxis and life satisfaction relate to each other, and then to interview H-MSM for an in-depth follow-up on survey concepts.

**Methods:**

Purposive sampling will be used to recruit men via online and offline venues. Data collection will be multifaceted and include an online questionnaire for adult cisgender men and a subsequent interview for H-MSM. The survey will be available in English, French, and Spanish. Structural equation modeling, underpinned by hegemonic masculinity and sexual script theories, will be performed to test the relationships among survey variables. Interpretive phenomenology will be employed on the qualitative data to consider how unique sociocultural factors influence the sexuality and experiences of H-MSM, allowing for similarities and differences across participants to be identified and explored.

**Results:**

Data collection began on November 26, 2024, and as of February 2025, data collection remains ongoing. We expect to conclude data collection and data cleaning by mid-summer 2025. Data analysis will begin in fall 2025. Our findings will provide a more nuanced understanding of the identity development, attraction, behavior, relationship negotiation, and technology use of H-MSM in comparison with GBQ+ men and concordant heterosexual men.

**Conclusions:**

This research aims to broaden the scope of existing literature and support advancements in interventions and knowledge to support the overall health and well-being of H-MSM. An examination of potential differences among H-MSM, concordant heterosexual men, and GBQ+ men aims to improve the understanding of H-MSM as a distinct population, without dismissing them as closeted GBQ+ men. This study aims to provide key insights into avenues for research and practice with men whose sexuality and sexual behaviors may be outside of commonly accepted norms.

**International Registered Report Identifier (IRRID):**

DERR1-10.2196/66897

## Introduction

### Background

Sexuality is neither fixed nor uniform, rather it is a multidimensional construct shaped by the following key components: identity, attraction, and behavior [1,2]. Sexual identity refers to both the label one uses to define their sexuality (eg, straight, gay, bi, pan, and fluid) and the social group status [3]. While sexual identity can change over time and develops in relation to contextual factors, including prescribed social norms and environment, it is often influenced by sexual attraction [3,4]. Sexual attraction, defined as the desire for sexual intimacy toward an individual or a group of people [5], serves as the primary foundation upon which one forms their sexual identity [4]. Together, sexual identity and sexual attraction work to motivate individuals’ sexual behaviors, referring to the sexual practices someone engages in both individually and with others [6].

Sexual identity, attraction, and behavior are often perceived as aligning with one another (ie, concordance), and for many individuals, they indeed do (eg, a cisgender man identifies as gay, is attracted to other men, and has sex with other men). Importantly, some individuals experience discordance among their identities, attractions, and behaviors (eg, a cisgender man identifies as straight, is attracted to men and women, and has sex with men). Specifically, studies have shown that some heterosexual men (ie, men who identify as being straight) are attracted to and engage in sex with other men [7,8].

Heterosexual-identified men who have sex with men (H-MSM) experience sexual identity and behavior discordance, and they are a concealed population group. A recent knowledge synthesis found that H-MSM may comprise 0.5%-3.5% of heterosexual men and 1.26%-5.4% of men who have sex with men (MSM) [8]. Unlike gay, bisexual, and queer (GBQ+) men and concordant heterosexual men, H-MSM exhibit incongruence between their identified sexual orientation and sexual activity. Health outcomes among H-MSM remain an underexamined area, although preliminary research suggests that they may experience unique disparities compared to both heterosexual men and openly gay or bisexual MSM [9]. Extant research conducted in the United States and Europe has found that H-MSM may feel greater guilt and shame, have less ability to cope, and have poorer sexual communication in their relationships than their gay and bisexual counterparts [9]. These factors introduce adverse physical and mental health outcomes for H-MSM, particularly with regard to shaping their views of self [9]. Previous research has demonstrated that H-MSM often view themselves as less happy compared to the average person and report receiving less social support than heterosexual men [9]. As such, H-MSM may benefit from specific interventions to support mental health–related outcomes, including stress reduction, increased support and resources, improved communication, and facilitated identity development, which would likely be most effectively mediated by internet-enabled and mobile (eg, smartphone) technologies [8,9]. Indeed, research shows that H-MSM frequently use internet-enabled and mobile technologies, partially because these platforms provide greater privacy compared to in-person venues [8]. Thus, there are possibilities that H-MSM will feel more comfortable with accessing support services via technology as such interventions may be more likely to protect their anonymity.

### Theoretical Foundations

Hegemonic masculinity and sexual script theories can be intertwined to help develop a stronger understanding of sexual identity congruence and incongruence among cisgender men. Both theories provide possible explanations for various sexual attitudes, self-expressions, and behaviors. More specifically, the theory of hegemonic masculinity states that maintaining masculine traits is culturally appealing and synonymous to what it means to be both heterosexual and a man [10]. According to this theory, adopting a sexual minority identity (eg, gay, bisexual, and queer) leads individuals to be perceived as less masculine and less worthy [10,11]. Similarly, the sexual script theory argues that sexual behaviors are socially constructed and are influenced by cultural, interpersonal, and intrapersonal pressures on how men should act [12,13]. That is, individuals navigate around attitudes toward sex, sexuality, and gender to determine how they will interact with others in different sexual situations. For example, men are expected to hold sexual agency and be sexually dominant and sexually motivated [14]. Some recent research suggests that heterosexual men do not always approve of these gendered sex scripts, yet they may still comply with them [15,16].

However, H-MSM tend to embrace hegemonic masculinity and traditional sex scripts to avoid being labeled as GBQ+, to maintain their heterosexuality, and to be perceived as respectable [9]. Men who identify as GBQ+ often find themselves challenging hegemonic masculinity and gendered sex scripts [17]. Thus, H-MSM may be more likely than both concordant heterosexual men and GBQ+ men to uphold higher levels of conformity to masculine norms, sexual identity confusion and exploration, and internalized homophobia. They may also have greater attraction to narcissism, a trait that has been associated with hypermasculinity [18]. Relatedly, there is emerging evidence that H-MSM enjoy discreet encounters and rough and aggressive sex with other men but emphasize relationships with women [9]. Perhaps heterosexual men feel pressured to treat women delicately, while MSM are more willing to be sexually adventurous. If so, there are possibilities that H-MSM have higher degrees of sexual sensation seeking, sexual compulsivity, and rough sex than concordant heterosexual men. However, only minimal research has examined these phenomena among all sexually active men to compare men of different identities and concordance statuses.

Moreover, scholars argue that traditional sex scripts instruct men to be hypermasculine and maintain sexual agency over their partners [14]. As such, regardless of sexual identity, embracing hegemonic masculinity and traditional sex scripts, characterized by upholding hypermasculine traits, may prevent sexually active men from openly communicating about sex with their partners, which may have further ramifications for sexual satisfaction. Indeed, sexual communication has been linked to greater sexual satisfaction [19].

Technology use may also influence sexual and relationship communication and other sexual behaviors. Technology, for example, has been used to facilitate sexual interactions, especially among MSM [8,20,21]. Research has also shown that H-MSM enjoy meeting male sexual partners online as opposed to meeting them in-person at GBQ+ venues as the internet gives them greater privacy, allowing them to remain discreet [8]. Therefore, H-MSM may exhibit greater technology use related to sex-seeking than other sexually active men. However, questions remain on how technology use influences sexual communication and satisfaction. Research on the role of technology in sexual communication and satisfaction among sexually active men is necessary to identify possible pathways for improving sexual relationships.

Furthermore, hypermasculinity can be damaging toward help-seeking and well-being, contributing to adverse health outcomes [22]. For instance, previous research has found that endorsing more heterosexual self-presentation and experiencing greater sexual identity uncertainty can be barriers to pre-exposure prophylaxis (PrEP) adherence [23]. Indeed, scholars have concluded that men often perceive help-seeking, even related to sexually transmitted infection treatment and prevention, as a sign of weakness and as being incompatible with what it means to be a masculine man [22,24,25]. Help-seeking, though, can mitigate stress and improve life satisfaction [26]. Thus, areas of identity, attraction, and behavior associated with hypermasculinity could be connected to lower PrEP uptake, worsened attitudes toward PrEP, and lower life satisfaction. More research is needed to understand these relationships and to promote HIV prevention efforts among H-MSM and other sexually active men.

### Focus of This Study

This study extensively explores the identity development, attraction, behavior, relationships, and technology use of H-MSM compared to GBQ+ men and concordant heterosexual men. H-MSM are an understudied population, and little is known about their specific experiences regarding identity development, attraction, behavior, relationships, and technology use. Currently available health services, supports, and interventions might overlook H-MSM, potentially limiting their access to care, tailored sexual health resources, mental health support, and community-based programs that address their specific needs. As such, the objectives of this study are to (1) survey men (H-MSM, GBQ+ men, and concordant heterosexual men) in Canada, the United States, and the United Kingdom to gain greater insights into how demographics, identity development, attraction, behavior, technology use, relationship negotiation and communication skills, and PrEP and life satisfaction relate to each other and (2) interview H-MSM about the constructs measured in the survey. These data will help researchers identify potential priority areas affecting H-MSM and will be used to develop pilot psychosocial interventions focused on helping H-MSM. Including GBQ+ men and concordant heterosexual men in the study will be useful in drawing comparisons and further investigating the experiences of H-MSM.

### Research Questions

The research questions are as follows:

1. How do the demographics, identity development, attraction, behavior, technology use, relationship negotiation and communication, health, and well-being of H-MSM influence each other, and how do these variables differ between H-MSM and GBQ+ men as well as concordant heterosexual men?
2. How do identity development, attraction, and behavior predict the relationship negotiation and communication of H-MSM?
3. How does technology use moderate the relationship negotiation and communication of H-MSM, if at all?
4. How do identity development, attraction, behavior, relationship negotiation and communication, and technology use differ according to participant demographics?
5. How do H-MSM narratively conceptualize their identity development, attraction, behavior, relationship negotiation and communication, and technology use?

## Methods

### Study Design

This study uses a mixed-methods design employing both quantitative and qualitative research methods. The study is conducted virtually, and recruitment efforts span Canada, the United States, and the United Kingdom. Data are collected through an online survey as well as semistructured interviews.

### Study Team

The research team consists of 1 principal investigator, 6 co-investigators, 1 research coordinator, 3 graduate research assistants, and 2 community advisors. Members of the study team are also practicing psychotherapists.

### Ethical Considerations

This study has been approved by the University of Regina’s Research Ethics Board (approval number: #610). Informed consent will be obtained from each participant before their involvement in both surveys and interviews. More specifically, prior to participating in the survey, individuals will provide informed consent online. In addition, verbal consent will be obtained prior to beginning the interviews. Participants can skip any questions they do not wish to answer. Participants will be informed of their right to withdraw during the consent process and in the consent form. Participants are welcome to leave the interview at any time and to withdraw their interview data within 7 days of completing the interview. This is outlined in the consent form and will be explained in the consent process. Participants can also contact the principal investigator with any questions or concerns.

### Recruitment

Recruitment will be purposive and multipronged as H-MSM are hidden and difficult to reach [8]. We will advertise recruitment on MSM mobile apps (eg, Grindr, Scruff, and Squirt) and other social media apps (eg, Facebook, Instagram, and Snapchat) that are known to be successful for recruiting MSM. These advertisements will also be displayed in various venues that sexually active men may frequent, including sexually transmitted infection and public health clinics. GBQ+ men are more likely to participate in research when advertisements display affirming images, while H-MSM are more likely to avoid research when stereotypical caricatures of GBQ+ are displayed [8]. Thus, using multiple advertisements with diverse representation will help us in recruiting diverse participants and will ensure that we obtain a representative sample. The advertisements will contain the survey link, which includes the online consent form, and a weblink to Eaton Lab [27] for further details on the study.

People will be eligible to participate in the survey if they self-identify as a man and reside in Canada, the United States, or the United Kingdom. They also must be 18 years or older to participate and must be able to read fluently in English, Spanish, or French. Participants will be excluded if they do not identify as a man or do not reside in Canada, the United States, or the United Kingdom. They will be excluded if they are under 18 years of age or cannot read fluently in English, Spanish, or French.

To participate in the interview, individuals must indicate at the end of the survey that they are interested in participating in an interview. Additionally, participants must meet the above inclusion criteria and self-identify as a heterosexual, cisgender man who has had sex with at least one other man in their lifetime. Additionally, participants can only participate in an interview if they speak fluent English.

Multiple strategies will be used to identify and remove false responses or software robots, both representing common problems in internet-based research [28-30]. For example, a rapid succession of responses from the same IP address is one of the most common indicators that those responses are fraudulent [28]. Software robots also sometimes provide irrelevant answers to open-ended responses. Therefore, such cases will be removed prior to data analysis. Further, attention checks (eg, “Please select 4”) will be used throughout the survey to ensure that participants are real humans who are reading the questions and not blindly providing responses to enter the raffle for an incentive.

### Data Collection

Data collection will be multifaceted and include an online questionnaire and subsequent interview, dependent on participant eligibility.

#### Surveys

Upon agreeing to participate, participants will be directed to the online survey, which will be administered through Qualtrics. The survey is estimated to take approximately 30-45 minutes. The survey will be offered in English, French, and Spanish, depending on the participants’ preferences. The survey is divided into 12 sections: (1) screening, (2) demographics, (3) identity development, (4) attraction, (5) behavior, (6) coping with same-sex attraction, (7) relationship negotiation and communication, (8) technology use, (9) PrEP, (10) life satisfaction, (11) additional demographics, and (12) contact or follow-up information. The English survey questionnaire is provided in Multimedia Appendix 1. The study’s questionnaire is programmed through Qualtrics XM Software, Version 2019.

#### Interviews

Interviews will focus on developing a stronger understanding of the identity development, attraction, behavior, relationship negotiation and communication, and technology use of H-MSM and will be held via Zoom (Zoom Communications), which is an online video and audio meeting program. Participants will be given the option of phoning into the Zoom meeting or joining via a Zoom link. These Zoom meetings require either a phone-in and code to be entered or an account number and an individual login password. Therefore, individuals will be able to participate in the interviews from wherever they wish, although they are encouraged to be alone in a safe and quiet environment throughout the duration of the interview. Interview questions will relate to topics surrounding sex and sexuality, particularly participants’ sexual orientation, attraction, behavior, and relationships. The interview questionnaire is provided in Multimedia Appendix 2.

### Measures

A matrix of survey measures is provided in Multimedia Appendix 3.

#### Demographics

Participants will be asked to complete a demographics questionnaire created for this study. Questions pertain to age, gender identity, sex assigned at birth, sexual identity, sexual orientation, race, religion, marital status, and past sexual activity.

#### Identity Development

This study’s questionnaire uses 7 validated scales to assess constructs related to identity development, including sexual identity, sexual orientation beliefs, identity confusion, and internalized homophobia.

Sexual identity will be measured using 4 validated scales: Kinsey Scale [31], Heterosexual Self-Presentation Subscale of the Conformity to Masculine Norms Inventory-46 (CMNI-46; α=.90) [32], Measure of Sexual Identity Exploration and Commitment (α=.75-.93) [33], and Sexual Orientation Identity Development Scale (α=.61-.81) [13].

The Kinsey Scale will be used to examine differences in identity questions, as it captures the continuum of sexual experiences, demonstrating that sexuality cannot be easily categorized [31].

The Heterosexual Self-Presentation Subscale of the CMNI-46 [32] measures conformity to traditional masculine norms, a common ideal among H-MSM, and has shown good internal consistency. This subscale is relevant to this study as many H-MSM report not wanting to be perceived as gay. The CMNI-46 has been used to measure specific masculine norms within a sample of H-MSM [34,35] and can be used to measure conformity to specific masculine norms [36]. This particular subscale of the CMNI-46 relates to an individual’s sentiments regarding being perceived as gay and is pertinent to this study’s sample as many H-MSM report not wanting to be perceived as gay [9].

The Measure of Sexual Identity Exploration and Commitment is a self-report questionnaire designed to assess the complexities of sexual identity development and has been used to examine the processes of sexual identity formation across diverse populations, encompassing various sexual orientations, genders, and cultural backgrounds [31,37]. It consists of 22 items across 4 subscales: commitment, exploration, sexual orientation identity uncertainty, and synthesis or integration. These subscales provide greater data related to sexual identity development. Worthington et al [33] demonstrated construct validity, and Dillon et al [37] found that the exploration and commitment subscales were valid. Rosenberg [38] demonstrated criterion validity for H-MSM and others. Furthermore, both Dillon et al [37] and Worthington et al [33] demonstrated high internal consistency of subscales, with the latter also finding good test-retest reliability across 2-week intervals.

The Sexual Orientation Identity Development Scale [32], though not yet validated with H-MSM, has been used with plurisex Latinx youth [39]. The measure is adapted from the Brief Ethnic Identity Scale [40] and measures identity exploration, resolution, and affirmation. Higher scores suggest higher levels of sexual orientation identity exploration, resolution, and affirmation. Toomey et al [41] found strong convergent validity when relating constructs to internalized homonegativity and self-esteem among Latino/a LGBQ adolescents (age 14-24 years), as well as factorial validity in confirmatory factor analysis. Further, Toomey et al [41] found good internal consistency between items.

Sexual orientation beliefs will be measured by the Sexual Orientation Beliefs Scale (α=.75-.84) [42]. This instrument measures beliefs about sexual orientation and includes 4 subsets: naturalness (items 1-6), discreetness (items 7-12), homogeneity (items 13-23), and informativeness (items 24-31). Although not yet validated with H-MSM, Arseneau et al [42] found strong factorial validity, good test-retest reliability, and good internal consistency with heterosexual undergraduate students.

The final facets of identity development and confusion, and internalized homophobia will be measured using the Sexual Identity Confusion Scale (α=.89) [43,44] and the Shidlo-Revised Nungesser Homosexual Attitudes Inventory (α=.94) [45,46], respectively. The Sexual Identity Confusion Scale is a self-report measure designed to assess the degree of confusion individuals experience regarding their sexual identity. It captures the uncertainties and conflicts that may arise during the process of sexual identity development. Sandfort et al [44] reported good internal consistency. The Shidlo-Revised Nungesser Homosexual Attitudes Inventory is a self-report measure designed to capture attitudes toward homosexuality, particularly focusing on internalized homophobia among gay men. Shidlo [46] identified construct validity, and Nungesser [45] demonstrated good internal consistency with each factor. Factors include attitudes toward one’s own homosexuality, attitudes toward other homosexuals, and attitudes toward the disclosure of one’s own homosexual identity to others. The Sexual Identity Confusion Scale and the Shidlo-Revised Nungesser Homosexual Attitudes Inventory have not been validated in H-MSM.

#### Attraction

Four components of attraction, including current levels of sexual desire or interest, sexual preference, attraction, and intimacy, will be captured by the study’s questionnaire using items drawn from existing literature as well as validated scales.

Current levels of sexual desire or interest will be measured using 2 moderately correlated items (“How would you rate the degree of your current sexual interest?” and “How would you rate your current desire for sexual activity?”), which were previously used by Štulhofer et al [47] in a sample of men who had reported having exclusively or mostly male sexual partners in the past 5 years (*r_s_*=0.63; *P*<.01). Both items use a 5-point scale, ranging from 1 (very high) to 5 (very low). Scores are summed and reversed so that higher scores denote more sexual desire.

Sexual preference will be measured using the Sexual Preference Scale (=0.67; *P*<.01) [48], which measures attraction until age 15 years, current attraction, fantasies, and physical contact. It aims to capture the spectrum of sexual orientation, ranging from identified exclusive heterosexuality to identified exclusive homosexuality. The scale uses a numerical spectrum ranging from 0 (exclusively attracted to the same sex) to 100 (exclusively attracted to the opposite sex), increasing in increments of 10, to quantify an individual’s sexual orientation. McConaghy and Blaszczynki [48] reported physiological validation of the questionnaire by measuring penile volume.

The Attraction/Intimacy Inventory (α=.91-.99) [49] will be used to measure attraction or intimacy. The Attraction/Intimacy Assessment Inventory is a 20-item self-report measure designed to evaluate various aspects of interpersonal attraction and intimacy between individuals. The inventory has been used in various studies to explore relationships and has demonstrated good internal consistency, as indicated by Cronbach α coefficients reported in the literature [49]. Furthermore, the inventory’s test-retest reliability has been supported, making it a reliable tool for measuring attraction and intimacy over time.

Given the relationship between narcissism and hypermasculinity [18] and the valuation of hypermasculinity within hegemonic masculinity and traditional sex scripts [15,16], especially among H-MSM [9], examining attraction toward narcissism may be helpful for understanding differences across groups of men and understanding how certain forms of attraction influence relationships, community, and health. Specifically, narcissistic trait attraction will be measured using an unnamed scale previously used by Haslam and Montrose [50] in a study assessing narcissistic trait attraction among a sample of young adult heterosexual females. Haslam and Montrose [50] asked participants to consider 20 statements relating to the extent that they found narcissistic personality traits attractive in a potential mate. These 20 statements have been derived from the 40-item Narcissistic Personality Inventory and have been formatted to a Likert-type style. Accordingly, the scale is adapted from the Narcissistic Personality Inventory, which is a scale that is validated for use among nonclinical populations. To the best of our knowledge, this scale has not been used with H-MSM.

#### Behavior

Five facets of sexual behavior will be captured in the study’s questionnaire, including sexual satisfaction, coping with same-sex attraction, sexual sensation seeking, sexual compulsivity, and engagement in rough sex.

Sexual satisfaction will be assessed using 3 items previously used by Gonzaga et al [51] (α=.76) to measure this construct in a sample of romantic couples. Gonzaga et al [51] standardized and averaged the items to create a scale of sexual satisfaction. The items demonstrated good internal consistency and assessed: (1) how much participants agreed about sexual relations with their partner, (2) how enjoyable they found their sexual relations with their partner, and (3) how satisfactory they found the sexual relations with their partner. Similarly, coping with same-sex attraction will be measured using 46 items previously adopted in a study by Dehlin et al [52]. Specifically, in their study investigating sexual orientation change efforts in a sample of current or former members of the Church of Jesus Christ of Ladder-day Saints (LDS), Dehlin et al [52] asked participants which of several activities they had engaged in to “understand, cope with, or change” their sexual orientation. The first question generally asked if participants have or have not attempted a specific coping mechanism. For each coping strategy endorsed, 4 subsequent questions were asked: one related to the age they started the effort, another related to how long the effort lasted (in years), another related to the perceived effectiveness of each method, and another asking if they aimed to accept or change their sexual orientation. The authors reversed the scores for all the effectiveness items. These options were developed by the research team based on several sources, including direct clinical practice with LDS LGBTQ individuals and familiarity with LDS culture or practice and doctrine [53,54].

Sexual sensation seeking and sexual compulsivity will be measured using the Sexual Sensation Seeking Scale and the Sexual Compulsivity Scale (α=.75-.89) developed by Kalichman et al [55]. The former measures the degree to which one might seek new and exciting forms of sexual arousal [56], and the latter measures the extent of how compulsive one’s sexual thoughts and behaviors are [55]. The authors demonstrated convergent, divergent, and discriminant validity of the Sexual Sensation Seeking Scale in MSM, and showed that the scale had good internal consistency and test-retest reliability. Likewise, the authors demonstrated convergent, divergent, and discriminant validity of the Sexual Compulsivity Scale in MSM. This scale has been used in H-MSM; however, it has not been validated in this group specifically (eg, [57,58]).

Finally, attraction to rough sex will be measured in this study through items adapted from measures previously used by Hebernick et al [59]. Four items were used in the original study by Hebernick et al [59] to assess participants’ attraction to rough sex in a sample of undergraduate students. The measures involved single items and not a scale, indicating that a scale for attraction to rough sex should be created. These items have only been assessed as individual items. These 4 items have been adapted to fit this study, resulting in 28 additional items. The research team will consider conducting an exploratory factor analysis and confirmatory factor analysis to examine the structure of these items when combined to measure overall attraction to rough sex. 

#### Relationship Negotiation and Communication

Relationship negotiation and communication will be assessed by measuring heterosexual scripting and sexual communication. The Heterosexual Script Scale (α=.88) [60] will be used to measure heterosexual scripting, and the Sexual Communication Scale (α=.83-.96) [61] will be used to measure sexual communication.

The Heterosexual Script Scale measures the endorsement of sexual double standards, courtship strategies, and commitment strategies. It includes 4 subscales: courtship and commitment subscale, men as powerful initiators subscale, men value women’s appearance subscale, and sex defines masculinity and women set sexual limits subscale. Seabrook et al [60] reported discriminant validity of the scale’s distinct subscales, and construct validity was demonstrated by showing that the scale is correlated with (but distinct from) traditional gender role attitudes, traditional sexual scripts, sexism, self-objectification, and self-sexualization among undergraduate students.

The Sexual Communication Scale [62] measures the frequency of bidirectional communication, ease of own communication, and ease of partner’s communication. Moazami et al [61] found that the scale had good factorial validity and internal consistency among undergraduate students. For our purposes, the scale has been adapted, and 2 versions have been created: one for communication with men (12 items) and one for communication with women (12 items). A total partner sexual communication score can be computed by adding the scores of all 12 items. 

#### Technology Use

Seven categories of technology use will be measured in this study, including incidental information acquisition frequency, internet use levels, social media use, intent to use dating apps, attitude toward looking for romantic partners via dating apps, privacy settings, and Grindr use. We could not find measures on technology use validated for the study population. Therefore, we have selected items from extant research on closely related topics.

Incidental information acquisition frequency will be measured with 3 items that have been used to measure nonpurposeful information acquisition related to HIV/AIDS (α=.798) [63]. Venoit et al [63] found good reliability of these items in their original study with a sample of young MSM. In addition, internet use levels will be measured using 6 items used by Venoit et al [63] in the same study. A total of 13 items developed by the last author (LBM) via a survey of how sexual and gender diverse youth experience online negativity will measure how often individuals are online and use different apps on a regular basis.

Intent to use dating apps and attitude toward looking for romantic partners via dating apps will be measured using items that have previously been developed and used in a study to capture these behaviors and attitudes among participants (α=.79-.80) [63]. Chan [64] used 8 items to measure intent to use dating apps to look for romantic relationships and casual sexual relationships, and 6 items to measure attitudes toward using dating apps to look for romantic partners and attitudes toward looking for casual sexual partners via dating apps. All of the items were developed for the original study by Chan [64] and demonstrated strong internal consistency.

Grindr use will be measured using items previously used by Rice et al [65]. In a sample of young MSM, Rice et al [65] developed and administered a series of 10 items aimed to assess participants’ Grindr use patterns. Items included questions, such as how frequently they logged on to the app, the length of time they had been a Grindr user, the content of their profile photo, the time and day of typical use, concurrent substance and Grindr use, and motivations for using Grindr.

Lastly, participants will be asked to answer 5 single items to measure their personal privacy settings and the presence of different privacy settings across their technological use. All items are binary and have been developed specifically for this study.

#### PrEP Assessment

This study will measure participants’ uptake, knowledge, attitudes, and stigma regarding HIV PrEP. One item assesses whether participants currently take PrEP. To assess participants’ knowledge, attitudes, and stigma surrounding PrEP, this study uses items previously developed and used by Walsh [66]. A total of 13 items used by Walsh [66] to measure knowledge of PrEP in a sample of MSM will be used in this study. Walsh [66] reported good confirmatory factor analysis model fit and internal reliability (α=.90). Participants’ attitudes toward PrEP will be assessed using 5 items previously employed by Walsh [66] for this purpose. Walsh [66] reported good confirmatory factor analysis model fit and internal validity (α=.79), and Mueses-Marín et al [67] found good construct validity among Columbian MSM.

#### Life Satisfaction

Because hypermasculinity can be damaging to well-being [23] and because sexual communication and health are associated with improved life satisfaction [62,68-71], this study will include a measure of life satisfaction. Specifically, this study will measure participants’ life satisfaction using the Satisfaction with Life Scale [71], which has been used extensively in MSM. The scale consists of 5 items and has demonstrated high internal consistency and strong test-retest reliability (α=.87) [71].

### Analytical Plan

Data analysis will leverage a multimethod approach, integrating both quantitative and qualitative techniques.

#### Proposed Quantitative Analysis

Using Mplus version 8.9 [72], structural equation modeling (SEM) will be performed to test the relationships among the study variables. Models will be underpinned by hegemonic masculinity and sexual script theories and will be used to assess if and how measures of sexual identity, attraction, and behavior predict relationship negotiation and communication, and if and how these relationships are moderated by technology use. SEM will also be used to predict PrEP uptake and life satisfaction. The means and variance-adjusted weighted least squares estimator will be used to accommodate ordinal data for mediation [73]. In accordance with the best practices within SEM, both the measurement and structural models will be evaluated. First, the measurement model will be evaluated with confirmatory factor analysis to ensure the items adequately load onto their respective latent variables. Subsequently, control variables and paths between latent variables will be added to test the structural model. Then, the identification of the structural model will be confirmed before evaluating its model fit.

Model fit of both the measurement and structural models will be compared to prespecified criteria. For example, good model fit will be indicated by a nonsignificant *χ*^2^ result, a comparative fit index and Tucker-Lewis index of ≥0.95, a root mean square error of approximation of ≤0.06, and a standardized root mean square residual of ≤0.08 [74-77]. Modifications to each model will be considered one at a time and will be based on theory and extant research. Alternative models will be compared. The parameters will be evaluated once model fit is achieved.

#### Proposed Qualitative Analysis

Audio files will be uploaded to NVivo (Lumivero), a qualitative research software, which will be used to transcribe the interviews verbatim. Members of the research team will then check the quality of the transcription and provide edits as appropriate. Data from the interviews will be analyzed using interpretive phenomenological analysis (IPA), which is encouraged for use with marginalized communities, such as H-MSM, as it emphasizes contextual understanding [77]. IPA focuses on the participants and considers how unique sociocultural factors influence their experiences, allowing for similarities and differences across participants to be identified and explored [78]. To maintain the focus on participants and critically examine the similarities and differences across their stories, findings will focus on sharing and not generalizing their experiences [79].

To identify themes from the data, guidelines for IPA coding will be followed [78]. First, the researchers will immerse themselves in the data by reading and rereading the transcripts multiple times. After becoming familiar with the data, coders will record initial comments or codes related to content, context, symbols, metaphors, and other important aspects of language. During this stage, coders will also record their initial interpretations of participants’ stories. Throughout this process, the coding team will meet to reflect on initial thoughts, address any questions and concerns, acknowledge biases, and share feedback on effective coding strategies. Then, each coder will independently organize all codes into preliminary themes and subthemes. All members of the coding team will then meet to reflect on the similarities and differences between each coder’s organization of the codes. In this meeting, the coding team will finalize the themes and subthemes by reaching a consensus. Descriptions of each theme and subtheme will then be prepared and reported with exemplar quotes from the participants.

Several strategies will be employed to strengthen the trustworthiness of the study. For example, the inclusion of H-MSM as well as clinicians with relevant expertise throughout each stage of the study will help ensure that the study is meaningful and the findings are redistributed to relevant audiences. In addition, multiple coders, memo writing, an audit trail, peer feedback, and member checking will be used to strengthen the study.

## Results

Data collection began on November 26, 2024, and as of February 2025, data collection remains ongoing. As of February 2025, we have enrolled 231 participants, including 25 H-MSM, 158 GBQ+ MSM, and 48 concordant heterosexual men. We expect to conclude data collection by mid-summer 2025. Subsequently, we will begin analyses. We expect that the findings will provide a more nuanced understanding of the identity development, attraction, behavior, relationship negotiation, and technology use of H-MSM in comparison to GBQ+ MSM and concordant heterosexual men.

## Discussion

### Principal Results

Findings from this study will contribute to a more comprehensive understanding of how identity, attraction, and behavior interact within different subgroups of men. By exploring these outcomes, we hope to generate knowledge that might inform future interventions aimed at supporting the health and well-being of H-MSM.

### Comparison With Prior Work

This is the first comparative study of men’s sexuality that will intentionally sample and focus on H-MSM to ensure that they are well represented in the study while also including concordant heterosexual men and GBQ+ men. Many previous studies have not accounted for sexual identity-behavior discordance [80], and other studies recruited MSM and only happened to include H-MSM [80]. Meanwhile, other studies intentionally examined sexual identity-behavior discordance but did not oversample H-MSM [81]. Moreover, most studies of H-MSM used qualitative designs [7]. Thus, this study will build upon the existing research by recruiting large samples of H-MSM, concordant heterosexual men, and GBQ+ MSM to investigate the similarities and differences among men having different sexualities, behaviors, and attractions.

An examination of potential differences among H-MSM, concordant heterosexual men, and GBQ+ MSM aims to improve the understanding of H-MSM and encourage the need to include them in further research and practice without dismissing them as closeted GBQ+ men [8,9]. Indeed, knowledge of differences among H-MSM, concordant heterosexual men, and GBQ+ MSM may strengthen arguments for specifically targeting H-MSM in future research and public health interventions. To this end, affirming sexual identities, regardless of concordance or discordance with sexual behaviors and attractions, may play a role in encouraging help-seeking related to health services. As such, this study seeks to support the normalization and validation of H-MSM, an understudied population in need of greater attention.

This study also provides opportunities to examine how identity development, attraction, behavior, and technology use influence relationship negotiation and communication, an area of sex research in need of greater attention. In fact, research with H-MSM has not previously considered sexual communication. Nonetheless, healthy sexual communication is critical for greater sexual satisfaction [19], which is linked to greater life satisfaction [62,68-71]. Thus, this study will explore factors that may affect sexual communication among H-MSM, offering potential insights to consider when designing interventions targeted toward promoting healthy sexual behaviors in H-MSM.

Further, this study will examine possible predictors of PrEP uptake among H-MSM. Previous research on PrEP uptake centered on the voices and experiences of GBQ+ MSM and only included H-MSM who were labeled as sex workers [82,83]. Therefore, this study seeks to extend the knowledge base of PrEP and HIV prevention to H-MSM with the hope of informing the means of facilitating health-seeking behaviors and improving health outcomes. Such evidence could highlight directions for future research and practice related to encouraging PrEP use to prevent the spread of HIV.

### Strengths and Limitations

This study has several methodological strengths. First, this study provides a large-scale, comprehensive examination of identity development, attraction, behavior, relationships, and technology use among H-MSM compared to GBQ+ men and concordant heterosexual men. Second, the study leverages both quantitative and qualitative methodologies. Third, the study employs multiple recruitment strategies to reach a diverse sample of participants across Canada, the United States, and the United Kingdom. This research will broaden the scope of existing literature and contribute to advancements in interventions and knowledge to support the overall health and well-being of H-MSM.

This study design has several limitations. As a cross-sectional study, causality will not be established. The lack of repeated measures and probability sampling will limit the consistency and generalizability of the findings. Nonetheless, this study will be able to draw theoretically-driven comparisons to support the design of future interventions and investigations.

### Future Directions

Findings from this study are positioned to support future research and intervention development for H-MSM, a historically underserved and hard-to-reach population. Future studies may wish to address the limitations of this study, particularly by using longitudinal designs to assess changes over time and employing probability sampling to improve generalizability.

We hope to disseminate our findings through multiple channels, particularly given the relevance for both academic and community-based settings. These include conference presentations, knowledge mobilizations targeting community-based sexual health services, and peer-reviewed publications.

## Appendix

Multimedia Appendix 1 English survey questionnaire.

Multimedia Appendix 2 Interview questionnaire.

Multimedia Appendix 3 Survey matrix.

Multimedia Appendix 4 Peer-review reports by the Education and social work (12B) Committee, Insight Grants, Social Sciences and Humanities Research Council of Canada (SSHRC).

## Abbreviations

CMNI-46: Conformity to Masculine Norms Inventory-46
GBQ+: gay, bisexual, and queer
H-MSM: heterosexual-identified men who have sex with men
IPA: interpretive phenomenological analysis
LDS: Church of Jesus Christ of Ladder-day Saints
MSM: men who have sex with men
PrEP: pre-exposure prophylaxis
SEM: structural equation modeling

## Data Availability

The datasets generated or analyzed during this study will be available on Borealis once data collection and cleaning are complete.
