# Supplementary material for: Assessment of Heterosexual-Identified Men Who Have Sex With Men and Men of Diverse Sexual Identities: Protocol for an International, Multilingual, Online, Comparative Sexuality Study
Source: JMIR Res Protoc. 2025 Apr 30;14:e66897. doi: 10.2196/66897 (PMC12079075; doi:10.2196/66897)
Supplement: Multimedia Appendix 1 [file resprot_v14i1e66897_app1.docx]

Multimedia Appendix 1. English survey questionnaire.

Start of Block: Language Selection

LanguageSelection Please select your preferred language using the drop-down menu located in the top-left corner of this page.

Veuillez sélectionner votre langue préférée en utilisant le menu déroulant situé dans le coin supérieur gauche de cette page.

Seleccione su idioma preferido utilizando el menú desplegable ubicado en la esquina superior izquierda de esta página.

End of Block: Language Selection

Start of Block: LOIandConsent

LOI1 **Project Title: Men’s Sex Survey**

 **Introduction**

 Before agreeing to participate in this research study, it is important that you read and understand this research consent form. This form presents the information we think you need to know about this study. With this information, you can decide whether you want to be in the study. If you have questions after you read this form, please ask one of the people working on the study. You should not sign this form until you are sure you understand everything on it. You may also want to talk about participating in this study with a family member or a close friend. We encourage you to save a copy of this consent form for your own records. You can do so by selecting the print button your web browser.             

 **Researchers**

 Dr. Andrew D. Eaton (Associate Professor, Faculty of Social Work – Saskatoon Campus, University of Regina, Andrew.eaton@uregina.ca, 306-664-7371)

 Mr. Travis R. Scheadler (PhD Student, The Ohio State University, College of Social Work, Scheadler.2@osu.edu)

 Dr. Paul Shuper (Senior Scientist, Centre for Addiction and Mental Health, Assistant Professor, University of Toronto, paul.shuper@camh.ca)

 Dr. Lauren B. McInroy (Associate Professor, The Ohio State University, College of Social Work, mcinroy.1@osu.edu)

 Dr. Frank R. Dillon (Professor, Arizona State University, frank.dillon@asu.edu)

 Mr. Adam Busch (Adam Busch Therapy, adambuschtherapy@gmail.com)

 Dr. Tyrone Curtis (University of Victoria, tcurtis@uvic.ca)

 Mr. Daniel Vandervoort (University of Regina, d.vandervoort@utoronto.ca)  

 Ms. Megan Rowe (University of Regina, mg271665@dal.ca) 

 Mr. Salem Rao (University of Regina, salem.rao@mail.utoronto.ca) 

 **Purpose(s) and Objective(s) of the Research**

 We are inviting you to participate in this research study to understand your experiences related to your sexual identity development, behaviours, attractions, relationships, and technology use. In particular, we want to gain greater insight into the lived experiences of heterosexual men who have sex with men, with the goal of determining directions for targeted intervention efforts to promote healthy lives. 

 We will present this study at juried conferences and we intend to write articles about this study for peer-reviewed journals. We also plan to create community reports, conference presentations, plain language summaries, blogs and media reports. These publications will not include any personally identifiable information. Notice of these publications will be posted on the University of Regina’s Faculty of Social Work’s website.

 **Eligibility**
 If you are interested in participating, we welcome your participation in this project if you:
 •    Are a cisgender man
 •    Are at least 18 years old or older
 •    Reside in Canada, the United States, or the United Kingdom

 **Procedures**

 If you consent to participate in this study, your participation will involve completing an online survey that is estimated to take 30-45 minutes. You will be given a list of resources specific to men who have sex with men and crisis resources at the end of the survey. 

 During the survey, you may be asked if you are interested in participating in an interview. If you indicate interest in participating in an interview and you are a heterosexually-identified man who has sex with other men, a member of the research team may reach out to you via email to schedule an interview over Zoom. During the Zoom meeting, the researcher will review this consent form and you may ask any questions that you may have. That meeting will also include the interview, if you consent to participate. The interview is estimated to last approximately 60-90 minutes. 

 You do not have to answer any question that you do not feel like answering during the survey or interview. You do not have to give a reason for not answering if you do not want to. You can also withdraw your consent from the study during the survey or interview, and for one week following completion of the interview with no consequence. Withdrawing from the study will not affect your relationship with any of the study investigators, your relationship with any of the investigators’ institutions or organizations, nor will it affect your eligibility to participate, volunteer, and/or work in future research projects offered by the investigators and/or their affiliations. 

 Please feel free to ask any questions regarding the procedures and goals of the study or your role.

 **Funded by**

 This study is funded by the Social Sciences and Humanities Research Council (SSHRC) in Canada. 

 **Potential Risks**

 There are no known or anticipated risks to you by participating in this research, but there is a chance that you could find some parts of this study uncomfortable. You are going to be asked some questions about your identity development, attraction, behaviour, relationships, and technology use. We need to ask these questions for the study to understand how best to provide support in these areas. 

 If you feel any discomfort during the study, we will encourage you to follow up with Dr. Eaton (Principal Investigator), who can refer you to local support services. 

 Once seven days have passed since interview completion, the principal investigator will de-identify your data by deleting any identifiable information. Only the principal investigator and research coordinator will have access to identifiable study data. The rest of the research team will only have access to the de-identified data. 

 Your name or anything else that identifies you specifically will not be included when we write up the results. 

 During your participation in the interview portion of the study, you can stop being part of the study and choose to have the information already collected from you included in the study deleted. If you choose to withdraw after one week following completion of the interview, we will not be able to delete your information as it will have been de-identified. Your ability to access any services or resources from the affiliated institutions will not be impacted by either being in the study or withdrawing from the study at any time. 

 There are inherent security issues when using email as a communication tool. By default, emails are not encrypted and are vulnerable to interception by outside sources or someone may see that you are involved in this research if you leave your browser open. We will use the term/phrase “Men’s Health Study” in the subject-line of all email correspondence so you will know the email is from us and recommend you submit any email queries using the same term.

 The data collected in this study are strictly confidential. No identifying information will be collected at any time. We will treat your personal information as confidential although absolute privacy cannot be guaranteed. Please do not put your name or other identifying information in the questionnaire.

 We collect data through the software Qualtrics, which uses servers with multiple layers of security to protect the privacy of the data (e.g., encrypted websites and password protected storage). Your data will be stored and protected by Qualtrics on Ireland- based servers but may be disclosed via a court order or data breach.

LOI2 **Potential Benefits**

 You may not benefit directly from participating in this research. We hope that this study will help improve services for heterosexual men who have sex with other men, and other sexually active men, in Canada, the United States, and the United Kingdom. 

 **Compensation**

 To compensate for your participation in the survey, you will be asked if you would like to enter into a raffle to win a $30 electronic Amazon gift card. There will be 30 gift cards available to be raffled among participants from Canada, 30 gift cards available to be raffled among participants from the United States, and 30 gift cards to available to be raffled among participants from the United Kingdom. If you withdraw participation during the survey, you will still be eligible to enter the raffle. The gift card will be sent to your email. 

 To compensate for your participation in the interview, you will be provided with a $30 electronic Amazon gift card after completing the interview. If you withdraw your participation during the interview, you will still receive the $30 as compensation. The gift card will be sent to your email. 

 **Confidentiality**

 All information collected in this study is confidential, which means that the study team will not tell other participants, faculty, staff, or anyone else what you say in the study. 

 In order to comply with University policy regarding gift card compensation, your email address will be shared with Financial Services at the University of Regina. This is the only information in that will be shared. All email communications with you will not contain any information about the study and will be deleted immediately upon receipt of the form. There are inherent security issues when using email as a communication tool. By default, emails are not encrypted and are vulnerable to interception by outside sources or someone may see that you are involved in this research if you leave your browser open. We will use the term/phrase “Men’s Health” in the subject-line of all email correspondence so you will know the email is from us.”

 The Research Team will keep all of your answers confidential to the extent permitted by law. There are certain situations where we would not be able to keep everything you say confidential, due to legal limitations: 1) if you are at imminent risk of committing suicide or inflicting severe bodily harm on another person, 2) if you told us about a child under the age of 16 at risk of neglect, and 3) if your records were subpoenaed by a Canadian governmental organization. A quote from your interview participation may be used when the study’s results are published; no identifiable information will be included in the quote, however, we cannot guarantee complete anonymity.

 The only individuals who may see your name are Dr. Eaton (Principal Investigator) and Mr. Travis Scheadler (research coordinator). We will never give out your name to anyone else unless a court of law forces us to do so. 

 **Storage of Data:**  

 We will store your de-identified data from the survey and interview on a password-protected computer at the University of Regina’s Faculty of Social Work and/or a password-protected computer at The Ohio State University College of Social Work. Your name and other information that could tell others who you are will not be stored. All identifiable survey data (e.g., email address) will be separated from the rest of your data for the raffle and all survey data will then be aggregated. All identifiable interview data will be destroyed after the de-identified transcript has been created. Contact information for participants will be saved in a master file that will be password-protected, encrypted, and saved in a separate folder. The master file will be deleted after the completion of data analysis, which is expected to occur within one year of completing data collection. Any remaining file will be destroyed 7 years after completion of the study.  

 Your contact information will be electronically destroyed after data analysis has been completed and all incentives have been delivered. 

 **Right to Withdraw**

 Your participation is voluntary and you can answer only those questions that you are comfortable with.  You may withdraw from the research project for any reason, at any time without explanation or penalty of any sort.

 Whether you choose to participate or not will have no effect on your position [e.g. employment, class standing, access to services] or how you will be treated.

 Please note that you will not be able to withdraw from the survey portion of the study because no identifiable information will be collected. Therefore, there will be no way to identify you and remove your data. 

 Should you wish to withdraw during the interview portion of the study or within 7 days of participating in the interview, your data will be electronically destroyed. 

 Your right to withdraw data from the interview portion of the study will apply until 7 days have passed from your participation. After this date, it is possible that some results have been analyzed, de-identified, written up and/or presented and it may not be possible to withdraw your data.

 **Follow up**

 We anticipate publishing the results in the spring of 2026. 

 To obtain results from the study, please contact Dr. Andrew Eaton (Principal Investigator) at Andrew.eaton@uregina.ca or visit the University of Regina’s Faculty of Social Work’s website for more information. 

 The Faculty of Social Work at University of Regina is located at 111-116 Research Dr, Saskatoon, SK, CA S7N 3R3.

 **Questions or Concerns**

 Contact the researcher(s) using the information at the top of page 1.

 This project has been approved on ethical grounds by the University of Regina Research Ethics Board on (insert date).  Any questions regarding your rights as a participant may be addressed to the committee at (306-585-4775 or research.ethics@uregina.ca).  Out of town participants may call collect.

 Please note that contacting the researcher(s) with questions about your participation in the study will result in a breach of your confidentiality. Specifically, you contacting the researcher(s) about your participation in the study will allow the researcher(s) to know your identity.

 **Consent**
 **Continued or On-going Consent**

 If you participate in a follow-up interview, the researcher will review this consent form with you prior to the start of the interview. You will have the opportunity to ask any questions you may have. The researcher will then ask for your oral consent to proceed with the interview. The interview will occur on Zoom at an agreed upon time. 

 Finally, you are encouraged to be alone and in a safe and quiet environment throughout the duration of the interview to prevent others from hearing your responses. It is also advisable to be alone and in a safe and quiet environment throughout the survey to prevent others from seeing your responses. 

| 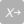 |
| --- |

ConsentSelection **IMPLIED CONSENT FOR SURVEYS**
 By completing and submitting the questionnaire, **YOUR FREE AND INFORMED CONSENT IS IMPLIED** and indicates that you understand the above conditions of participation in this study. Please select your choice:

- I CONSENT to participating in this study (1)
- I DO NOT CONSENT to participating in this study (2)

Skip To: End of Survey If IMPLIED CONSENT FOR SURVEYS By completing and submitting the questionnaire, YOUR FREE AND INFORME... = I DO NOT CONSENT to participating in this study

End of Block: LOIandConsent

Start of Block: Screening Questions

| 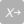 |
| --- |

gend0 Do you identify as a man?

- Yes (1)
- No (2)

Skip To: End of Survey If Do you identify as a man? = No

| 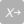 |
| --- |

age0 Are you over the age of 18?

- Yes (1)
- No (2)

Skip To: End of Survey If Are you over the age of 18? = No

| 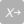 |
| --- |

Q143 What country do you live in?

- Canada (1)
- United Kingdom (3)
- United States (2)
- None of the Above (999)

Skip To: End of Survey If What country do you live in? = None of the Above

End of Block: Screening Questions

Start of Block: Demographics

Age How old are you (in years?)

________________________________________________________________

| 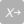 |
| --- |

Gender Please select your gender identity. Choose as many of the labels from the list as you feel apply to you.

- Agender (1)
- Cisgender man (2)
- Cisgender woman (3)
- Gender expansive (4)
- Genderfluid (5)
- Gender non-conforming (6)
- Man (7)
- Woman (8)
- Transgender (9)
- Transgender man (10)
- Transgender woman (11)
- Two-Spirit (12)
- Nonbinary (13)
- My gender identity is not represented in this list. I would describe my gender identity as: (0) __________________________________________________
- I prefer not to say. (999)

| 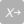 |
| --- |

Gender Many people use multiple terms to describe their gender identity. However, it may not be possible to include all unique combinations in each of our analyses. Therefore, if you had to select only one, which would you choose to BEST represent your gender identity?

- Agender (1)
- Cisgender man (2)
- Cisgender woman (3)
- Gender expansive (4)
- Genderfluid (5)
- Gender non-conforming (6)
- Man (7)
- Woman (8)
- Transgender (9)
- Transgender man (10)
- Transgender woman (11)
- Two-Spirit (12)
- Nonbinary (13)
- My gender identity is not represented in this list. I would describe my gender identity as: (0) __________________________________________________
- I prefer not to say. (999)

| 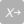 |
| --- |

Sex Please select the sex you were assigned at birth (i.e., what is listed on your birth certificate).

- Male (1)
- Female (2)
- Intersex (3)
- Other: (0) __________________________________________________
- I prefer not to say. (999)

| 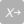 |
| --- |

Sexual orientation Please select your sexual orientation or identity. Choose as many of the labels from the list as you feel apply to you.

- Asexual (1)
- Bisexual (3)
- Demisexual (4)
- Gay (6)
- Heterosexual (7)
- Pansexual (8)
- Queer (9)
- Questioning/Unsure (10)
- Straight (11)
- Two-Spirit (12)
- My sexuality is not represented in this list. I would describe my sexuality as: (0) __________________________________________________
- I prefer not to say. (999)

| 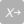 |
| --- |

Sexual orientation Many people use multiple terms to describe their sexuality. However, it may not be possible to include all unique combinations in each of our analyses. Therefore, if you had to select only one, which would you choose to BEST represent your sexual orientation.

- Asexual (1)
- Bisexual (4)
- Demisexual (5)
- Gay (7)
- Heterosexual (8)
- Pansexual (9)
- Queer (10)
- Questioning/Unsure (11)
- Straight (12)
- Two-Spirit (13)
- My sexuality is not represented in this list. I would describe my sexuality as: (0) __________________________________________________
- I prefer not to say. (999)

| 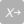 |
| --- |

Race Please select your race. Choose all that apply.

- White (1)
- Black (2)
- Hispanic (3)
- Latino/Latiné (4)
- Middle Eastern (5)
- Indigenous: First Nations, Inuit, or Métis (6)
- Indigenous: Native American (7)
- East Asian (8)
- North Asian (9)
- South Asian (10)
- Southeast Asian (11)
- Other: (0) __________________________________________________

| 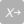 |
| --- |

Religion Which of the following best describes your religion?

- Agnostic (1)
- Atheist (2)
- Buddhist (3)
- Catholic (4)
- Christian (5)
- Hindu (6)
- Jewish (7)
- Muslim (8)
- Other: (0) __________________________________________________
- Prefer not to respond (999)

| 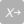 |
| --- |

Q12 What is your current relationship status?

- Single (1)
- Not married - open relationship/non-monogamous (2)
- Not married - monogamous relationship (3)
- Married - open relationship/non-monogamous (4)
- Married - monogamous (5)
- Divorced or Separated (6)
- Widowed (7)
- Other: (0) __________________________________________________
- Prefer not to respond. (999)

| 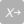 |
| --- |

Q15 Have you ever been sexually active with another man?

 Sexual activity is broad and includes not only **sexual intercourse but also physical intimacy with any sexual arousing, such as touching, holding, kissing, and close companionship.**

- Yes (1)
- No (2)

| 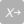 |
| --- |

sexwom Have you ever been sexually active with a woman?

 Sexual activity is broad and includes not only **sexual intercourse but also physical intimacy with any sexual arousing, such as touching, holding, kissing, and close companionship.**

- Yes (1)
- No (2)

Age We know you already shared your age in years, but to make sure that you are not a robot, please indicate which year you were born (such as 1990 or 2002).

________________________________________________________________

End of Block: Demographics

Start of Block: Identity Development

Display This Question:

If sexid1 = Heterosexual

Or sexid1 = Straight

Or sexid1 = Gay

| 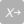 |
| --- |

kinsey Please rate which of the following best describes how you currently feel about yourself.

- 0: Exclusively heterosexual (0)
- 1: Predominantly heterosexual, incidentally homosexual (1)
- 2: Predominantly heterosexual, but more than incidentally homosexual (2)
- 3: Equally heterosexual and homosexual (3)
- 4: Predominantly homosexual, but more than incidentally heterosexual (4)
- 5: Predominantly homosexual, incidentally heterosexual (5)
- 6: Exclusively homosexual (6)

Display This Question:

If sexid1 = Heterosexual

And sexid1 = Straight

And sexid1 = Gay

| 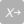 |
| --- |

hsp The following questions ask about how you present yourself. Please respond to each item using a scale from 1 (Strongly Disagree) to 4 (Strongly Agree).

|  | 1 - Strongly Disagree (1) | 2 - Disagree (2) | 3 - Agree (3) | 4 - Strongly Agree (4) |
| --- | --- | --- | --- | --- |
| Being thought of as gay is not a bad thing. (1) |  |  |  |  |
| I would be furious if someone thought I was gay. (2) |  |  |  |  |
| It would not bother me at all if someone thought I was gay. (3) |  |  |  |  |
| It would be awful if people thought I was gay. (4) |  |  |  |  |
| I would feel uncomfortable if someone thought I was gay. (5) |  |  |  |  |
| I try to avoid being perceived as gay. (6) |  |  |  |  |

| 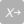 |
| --- |

siec The following items relate to your sexual needs, preferences, values, and identity. Please respond to each item using a scale from 1 (very uncharacteristic of me) to 6 (very characteristic of me).

|  | 1 - Very uncharacteristic of me (1) | 2 - Uncharacteristic of me (2) | 3 - Slightly uncharacteristic of me (3) | 4 - Slightly characteristic of me (4) | 5 - Characteristic of me (5) | 6 - Very characteristic of me (6) |
| --- | --- | --- | --- | --- | --- | --- |
| I have a firm sense of what my sexual needs are. (1) |  |  |  |  |  |  |
| I know what my preferences are for expressing myself sexually. (2) |  |  |  |  |  |  |
| I have never clearly identified what my sexual needs are. (3) |  |  |  |  |  |  |
| I have a clear sense of the types of activities I prefer. (4) |  |  |  |  |  |  |
| I do not know how to express myself sexually. (5) |  |  |  |  |  |  |
| I have never clearly identified what my sexual values are. (6) |  |  |  |  |  |  |
| I am actively trying new ways to express myself sexually. (7) |  |  |  |  |  |  |
| I can see myself trying new ways of expressing myself sexually in the future. (8) |  |  |  |  |  |  |
| I am open to experiment with new types of sexual activities in the future. (9) |  |  |  |  |  |  |
| I am actively experimenting with sexual activities that are new to me. (10) |  |  |  |  |  |  |
| I am actively trying to learn about my own sexual needs. (11) |  |  |  |  |  |  |
| My sexual values will always be open to exploration. (12) |  |  |  |  |  |  |
| I went through a period in my life when I was trying different forms of sexual expression. (13) |  |  |  |  |  |  |
| I went through a period in my life when I was trying to determine my sexual needs. (14) |  |  |  |  |  |  |
| I sometimes feel uncertain about my sexual orientation. (15) |  |  |  |  |  |  |
| My sexual orientation is not clear to me. (16) |  |  |  |  |  |  |
| My sexual orientation is clear to me. (17) |  |  |  |  |  |  |
| My sexual values are consistent with all of the other aspects of my sexuality. (18) |  |  |  |  |  |  |
| The sexual activities I prefer are compatible with all of the other aspects of my sexuality. (19) |  |  |  |  |  |  |
| The ways I express my self sexuality are consistent with all of the other aspects of my sexuality. (20) |  |  |  |  |  |  |
| My sexual orientation is compatible with all of the other aspects of my sexuality. (21) |  |  |  |  |  |  |
| My understanding of my sexual needs coincides with my overall sense of sexual self. (22) |  |  |  |  |  |  |

| 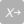 |
| --- |

soid The world is made up of people who have different sexual orientations. Sexual orientation refers to one’s romantic and sexual attractions to other people. Some examples of the sexual orientations that people may identify with include gay, lesbian, straight or heterosexual, bisexual, or queer. Using the rating scale provided, please chose the option that you believe best describes how you feel.

|  | 1 - Does not describe me at all (1) | 2 - Describes me a little (2) | 3 - Describes me well (3) | 4 - Describes me very well (4) |
| --- | --- | --- | --- | --- |
| I have attended events that have helped me learn about my sexual orientation, such as gay-straight alliance meetings or events at a lesbian, gay, bisexual, and transgender (LGBT) community center. (1) |  |  |  |  |
| I wish I were of a different sexual orientation. (2) |  |  |  |  |
| I understand how I feel about my sexual orientation. (3) |  |  |  |  |
| I know what my sexual orientation means to me. (4) |  |  |  |  |
| I have participated in activities that have taught me about my sexual orientation. (5) |  |  |  |  |
| I dislike my sexual orientation. (6) |  |  |  |  |
| I have a clear sense of what my sexual orientation means to me. (7) |  |  |  |  |

| 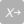 |
| --- |

atchk1 Please select number 4.

- 1 (1)
- 2 (2)
- 3 (3)
- 4 (4)

| 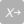 |
| --- |

sob The following items relate to your views on different sexual orientations. Please respond to each item using a scale of 1 (strongly disagree) to 5 (strongly agree).

|  | 1 - Strongly disagree (1) | 2 - Disagree (2) | 3 - Neither agree nor disagree (3) | 4 - Agree (4) | 5 - Strongly agree (5) |
| --- | --- | --- | --- | --- | --- |
| Sexual orientation is a category with distinct boundaries: A person is either gay/lesbian or heterosexual. (1) |  |  |  |  |  |
| People who identify as bisexual are confused about their true sexual orientation. (2) |  |  |  |  |  |
| Sexual orientation is a category with clear boundaries: A person is either gay/lesbian, bisexual, or heterosexual. (3) |  |  |  |  |  |
| It is possible to be “partially” or “somewhat” gay or straight. (4) |  |  |  |  |  |
| A person has only one true sexual orientation. (5) |  |  |  |  |  |
| People may reasonably identify as two sexual orientations at the same time. (6) |  |  |  |  |  |
| People who share the same sexual orientation pursue common goals. (7) |  |  |  |  |  |
| People with the same sexual orientation share a common fate. (8) |  |  |  |  |  |
| Individuals with the same sexual orientation seem to be connected to one another by some invisible link. (9) |  |  |  |  |  |
| People who have the same sexual orientation are very similar to one another. (10) |  |  |  |  |  |
| There are more similarities than differences among people who have the same sexual orientation. (11) |  |  |  |  |  |
| People who have the same sexual orientation interact frequently with one another. (12) |  |  |  |  |  |
| Individuals choose their sexual orientation. (13) |  |  |  |  |  |
| People have control over changing or keeping their sexual orientation. (14) |  |  |  |  |  |
| If someone comes out as gay or lesbian they were probably attracted to the same sex all along. (15) |  |  |  |  |  |
| It is impossible to truly change one’s sexual orientation. (16) |  |  |  |  |  |
| Sexual orientation is set early on in life. (17) |  |  |  |  |  |
| Sexual orientation is innate. (18) |  |  |  |  |  |
| Social and environmental factors are the main basis of an individual’s sexual orientation. (19) |  |  |  |  |  |
| The existence of different sexual orientations is natural. (20) |  |  |  |  |  |
| The idea that individuals have a “sexual orientation” is a social invention. (21) |  |  |  |  |  |
| Biology is the main basis of an individual’s sexual orientation. (22) |  |  |  |  |  |
| The percentages of people in different sexual orientation groups are roughly the same all over the world. (23) |  |  |  |  |  |
| Sexual orientation is an important characteristic of people. (24) |  |  |  |  |  |
| Knowing a person’s sexual orientation tells you a lot about them. (25) |  |  |  |  |  |
| It’s useful to group people according to their sexual orientation. (26) |  |  |  |  |  |
| A person’s sexual orientation is an important attribute. (27) |  |  |  |  |  |
| It is possible to know about many aspects of a person once you know her or his sexual orientation. (28) |  |  |  |  |  |
| If you don’t know a person’s sexual orientation, you can’t really say that you know that person. (29) |  |  |  |  |  |
| It is usually possible to know a person’s sexual orientation without being told. (30) |  |  |  |  |  |
| Most people view their sexual orientation as important to them. (31) |  |  |  |  |  |

| 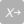 |
| --- |

confu The following items relate to how sure you are about your sexuality. Please respond to each item using a scale from 1 (strongly disagree) to 6 (strongly agree).

|  | 1 - Disagree strongly (1) | 2 - Disagree (2) | 3 - Somewhat disagree (3) | 4 - Somewhat agree (4) | 5 - Agree (5) | 6 - Agree Strongly (6) |
| --- | --- | --- | --- | --- | --- | --- |
| I'm not totally sure what my sexual orientation is. (1) |  |  |  |  |  |  |
| I keep changing my mind about my sexual orientation. (2) |  |  |  |  |  |  |
| I get very confused when I try to figure out my sexual orientation. (4) |  |  |  |  |  |  |

Display This Question:

If sexid1 != Heterosexual

And sexid1 != Straight

And sexman != No

| 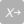 |
| --- |

inho The following items relate to your attitudes towards homosexuality. Please respond to each item using a scale from 1 (strongly agree) to 7 (strongly disagree).

|  | 1 - Strongly agree (1) | 2 - Agree (2) | 3 - Slightly agree (3) | 4 - Neutral (4) | 5 - Slightly disagree (5) | 6 - Disagree (6) | 7 - Strongly disagree (7) |
| --- | --- | --- | --- | --- | --- | --- | --- |
| When I am in a conversation with a gay/queer man and he touches me, it does not make me uncomfortable. (1) |  |  |  |  |  |  |  |
| When I think a lot about being gay/queer, I feel depressed. (2) |  |  |  |  |  |  |  |
| I am glad to be gay/queer. (3) |  |  |  |  |  |  |  |
| When I am sexually attracted to another gay/queer man, I feel uncomfortable. (4) |  |  |  |  |  |  |  |
| I am proud to be part of the gay/queer community. (5) |  |  |  |  |  |  |  |
| My homosexuality/queerness does not make me unhappy. (6) |  |  |  |  |  |  |  |
| Whenever I think a lot about being gay/queer, I feel critical about myself. (7) |  |  |  |  |  |  |  |
| I wish I were heterosexual/straight. (8) |  |  |  |  |  |  |  |
| I do not think I will be able to have a long-term relationship with another man. (9) |  |  |  |  |  |  |  |
| I have been in counseling because I wanted to stop having sexual feelings for other men. (10) |  |  |  |  |  |  |  |
| I have tried killing myself because I couldn't accept my homosexuality/queerness. (11) |  |  |  |  |  |  |  |
| There have been times when I've felt so rotten about being gay/queer that I wanted to be dead. (12) |  |  |  |  |  |  |  |
| I have tried killing myself because it seemed that my life as a gay/queer person was too miserable to bear. (13) |  |  |  |  |  |  |  |
| I find it important that I read gay/queer books or newspapers. (14) |  |  |  |  |  |  |  |
| It's important to me to feel part of the gay/queer community. (15) |  |  |  |  |  |  |  |
| Homosexuality/queerness is not as satisfying as heterosexuality/straightness. (16) |  |  |  |  |  |  |  |
| Homosexuality/queerness is a natural expression of sexuality in humans. (17) |  |  |  |  |  |  |  |
| Gay/queer men do not dislike women any more than heterosexual/straight men dislike women. (18) |  |  |  |  |  |  |  |
| Marriage between gay/queer people should be legalized. (19) |  |  |  |  |  |  |  |
| Gay/queer men are overly promiscuous. (20) |  |  |  |  |  |  |  |
| Most problems that gay/queer persons have come from their status as an oppressed minority, not their homosexuality/queerness per se. (21) |  |  |  |  |  |  |  |
| Gay/queer persons' lives are not as fulfilling as heterosexuals' lives. (22) |  |  |  |  |  |  |  |
| Children should be taught that being gay/queer is a normal and healthy way for people to be. (23) |  |  |  |  |  |  |  |
| Homosexuality/queerness is a sexual perversion. (24) |  |  |  |  |  |  |  |
| Adult gay/queer males who have sex with boys under eighteen years of age should be punished by law. (25) |  |  |  |  |  |  |  |
| I wouldn't mind if my boss knew that I was gay/queer. (26) |  |  |  |  |  |  |  |
| When I tell my straight friends about my homosexuality/queerness, I do not worry that they will try to remember things about me that would make me appear to fit the stereotype of a homosexual. (27) |  |  |  |  |  |  |  |
| When I am sexually attracted to another gay/queer man, I do not mind if someone else knows how I feel. (28) |  |  |  |  |  |  |  |
| When women know about my homosexuality/queer, I am afraid that they will not relate to me as a man. (29) |  |  |  |  |  |  |  |
| I would not mind if my neighbors knew that I ma gay/queer. (30) |  |  |  |  |  |  |  |
| It is important to me to conceal the fact that I ma gay/queer from most people. (31) |  |  |  |  |  |  |  |
| If my straight friends knew of my homosexuality/queerness, I would feel uncomfortable. (32) |  |  |  |  |  |  |  |
| If men knew about my homosexuality/queerness, I am afraid that they would begin to avoid me. (33) |  |  |  |  |  |  |  |
| If it were made public that I ma gay/queerness, I would be extremely unhappy. (34) |  |  |  |  |  |  |  |
| If my peers knew of my homosexuality/queerness, I am afraid that not many would want to be friends with me. (35) |  |  |  |  |  |  |  |
| If others knew of my homosexuality/queerness, I wouldn't worry particularly that they would think of me as effeminate. (36) |  |  |  |  |  |  |  |
| When I think about coming out to peers, I am afraid that they will pay more attention to my body movements and voice inflections. (37) |  |  |  |  |  |  |  |
| I am afraid that people will harass me if I come out more publicly. (38) |  |  |  |  |  |  |  |
| When I think about coming out to a heterosexua/straightl male friend, I do not worry that he might watch me to see if I do things that are stereotypically gay/queer. (39) |  |  |  |  |  |  |  |

hetde In your opinion, what does it mean to be heterosexual or straight?

________________________________________________________________

hetde What makes someone heterosexual/straight instead of gay or bisexual?

________________________________________________________________

| 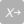 |
| --- |

surveycont. Do you want to continue taking this survey?

- Yes (1)
- I no longer wish to participate (2)

End of Block: Identity Development

Start of Block: Attraction

| 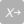 |
| --- |

clsd These next two questions ask about your sexual desires using a scale from 1 (very high) to 5 (very low).

|  | 1 - Very high (1) | 2 - High (2) | 3 - In the middle (3) | 4 - Low (4) | 5 - Very low (5) |
| --- | --- | --- | --- | --- | --- |
| How would you rate the degree of your current sexual interest? (1) |  |  |  |  |  |
| How would you rate your current desire for sexual activity? (2) |  |  |  |  |  |

| 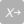 |
| --- |

pref The following items relate to your sexual preferences. Please respond to these items on a scale from 0 (exclusively attracted to the same sex) to 100 (exclusively attracted to the opposite sex).

|  | 0 - Exclusively attracted to the same sex (1) | 10 (2) | 20 (3) | 30 (4) | 40 (5) | 50 (6) | 60 (7) | 70 (8) | 80 (9) | 90 (10) | 100 - Exclusively attracted to the opposite sex (11) |
| --- | --- | --- | --- | --- | --- | --- | --- | --- | --- | --- | --- |
| Rate the degree to which, until the age of 15, you felt sexually attracted to members of the same sex as compared to those of the opposite sex on the following scale. (1) |  |  |  |  |  |  |  |  |  |  |  |
| Rate the degree to which you currently feel sexually attracted to members of the same sex as compared to those of the opposite sex on the following scale. (2) |  |  |  |  |  |  |  |  |  |  |  |
| Rate the degree to which in your current sexual fantasies you are aroused by members of the same sex as compared to those of the opposite sex, on the following scale. (3) |  |  |  |  |  |  |  |  |  |  |  |
| Rate the degree to which in physical contacts of any sort you have been conscious of sexual arousal to members of the same sex as compared to those of the opposite sex on the following scale. (4) |  |  |  |  |  |  |  |  |  |  |  |

| 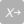 |
| --- |

Att/IntiAssInventory The following items relate to your attraction levels to males and females. Use the scale from 1 (does not describe me well) to 5 (describes me very well) to respond to each item.

|  | 1 - Does not describe me well (1) | 2 - Describes me a little (2) | 3 - Describes me somewhat (3) | 4 - Describes me well (4) | 5 - Describes me very well (5) |
| --- | --- | --- | --- | --- | --- |
| I know at least one female that I find physically attractive. (12) |  |  |  |  |  |
| I frequently notice attractive females. (13) |  |  |  |  |  |
| I have been sexually attracted to a female at least once. (14) |  |  |  |  |  |
| Females appear in my sexual fantasies. (15) |  |  |  |  |  |
| It is very hard for me to locate females who are interested in me sexually. (16) |  |  |  |  |  |
| I am in a romantic relationship with a male, and I feel he is very committed. (17) |  |  |  |  |  |
| I am in a relationship with a male to whom I am very committed. (18) |  |  |  |  |  |
| I am in a romantic relationship with a male, and neither of us is able to engage in sexual activities with people outside of our relationship. (19) |  |  |  |  |  |
| I am in a romantic relationship with a male. (20) |  |  |  |  |  |
| I am currently in a relationship with a male, and I would never cheat on him. (21) |  |  |  |  |  |
| I am in a relationship with a female to whom I am very committed. (22) |  |  |  |  |  |
| I am in a romantic relationship with a female, and I feel she is very committed. (23) |  |  |  |  |  |
| I am in a romantic relationship with a female who lets me know how she is feeling. (24) |  |  |  |  |  |
| I am in a romantic relationship with a female. (25) |  |  |  |  |  |
| I am in a romantic relationship with a female who does things just to show how much she cares. (26) |  |  |  |  |  |
| When I see an attractive male, I think of what it would be like to touch him. (27) |  |  |  |  |  |
| I masturbate while thinking about males. (28) |  |  |  |  |  |
| Males appear in my sexual fantasies. (29) |  |  |  |  |  |
| I frequently notice attractive males. (30) |  |  |  |  |  |
| I would enjoy some kind of sexual contact with an attractive male. (31) |  |  |  |  |  |

Display This Question:

If sexman = Yes

Or sexid2 = Bisexual

Or sexid2 = Gay

Or sexid2 = Pansexual

Or sexid2 = Queer

| 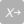 |
| --- |

Haslam&MontScale The following items relate to your attraction to particular qualities in men. Use the scale from 1 (strongly disagree) to 5 (strongly agree) when responding to each item.

|  | 1 - Strongly disagree (1) | 2 - Disagree (2) | 3 - Neither agree nor disagree (3) | 4 - Agree (4) | 5 - Strongly agree (5) |
| --- | --- | --- | --- | --- | --- |
| I am attracted to an egotistical male. (4) |  |  |  |  |  |
| An arrogant manner is attractive. (5) |  |  |  |  |  |
| I am attracted to a male who is preoccupied with brilliance and power. (6) |  |  |  |  |  |
| I am drawn to an attention seeking male. (7) |  |  |  |  |  |
| Confidence in a male is more alluring than modesty. (8) |  |  |  |  |  |
| A proud male is more appealing, than a passive male. (9) |  |  |  |  |  |
| Competitiveness is a desirable attribute in a male mate. (10) |  |  |  |  |  |
| Male vanity is an attractive attribute. (11) |  |  |  |  |  |
| A man who admires themselves is desirable. (12) |  |  |  |  |  |
| I prefer a male mate to have a more confident attitude than myself. (13) |  |  |  |  |  |
| A lack of empathy is desirable in a male. (14) |  |  |  |  |  |
| I am drawn to a male who uses manipulation to meet his needs. (15) |  |  |  |  |  |
| Having a self-important character is appealing. (16) |  |  |  |  |  |
| A male who seeks and expects praise is attractive (17) |  |  |  |  |  |
| A male who can influence people is attractive to me. (18) |  |  |  |  |  |
| I am attracted to men who take pleasure in being the centre of attention. (19) |  |  |  |  |  |
| I desire males who exhibit assertiveness. (20) |  |  |  |  |  |
| I am drawn to a male who displays authority. (21) |  |  |  |  |  |
| A male who is confident in displaying his body attracts me. (22) |  |  |  |  |  |
| A man who uses manipulation to influence his success at work is attractive. (23) |  |  |  |  |  |

| 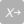 |
| --- |

surveycont. Do you want to continue taking this survey?

- Yes (1)
- I no longer wish to participate (2)

End of Block: Attraction

Start of Block: Behaviour

Sexual History In the last 12 months, approximately how many...

|  | 0 | 30 |
| --- | --- | --- |

| Men have you had sex with? () | 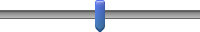 |
| --- | --- |
| Women have you had sex with? () | 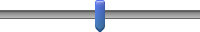 |
| Men have you had anal sexual intercourse with? () | 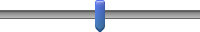 |
| Men have you had anal sexual intercourse with where you were the top/insertive partner? () | 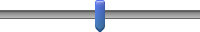 |
| Men have you had anal sexual intercourse with where you were the bottom/receptive partner? () | 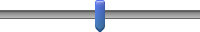 |
| Men have you had oral sexual intercourse with? () | 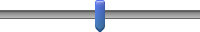 |
| Men have you gave oral sex to? () | 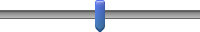 |
| Men have you received oral sex from? () | 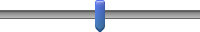 |
| Women have you had vaginal sexual intercourse with? () | 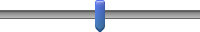 |
| Women have you had anal sexual intercourse with? () | 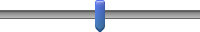 |
| Women have you had oral sexual intercourse from? () | 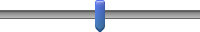 |
| Women have you performed oral sex on? () | 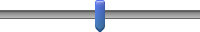 |

| 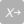 |
| --- |

satis1 How much do you and your partner(s) agree about your sexual relations?

- 1 - Always disagree (1)
- 2 - Often disagree (2)
- 3 - Sometimes disagree (3)
- 4 - Sometimes agree (4)
- 5 - Often agree (5)
- 6 - Always agree (6)

| 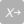 |
| --- |

satis2 How enjoyable are your sexual relations with your partner(s)?

- 1 - Not at all enjoyable (1)
- 2 - Slightly enjoyable (2)
- 3 - Moderately enjoyable (3)
- 4 - Enjoyable (4)
- 5 - Very enjoyable (5)

| 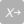 |
| --- |

satis3 How satisfied are you with your sexual relations with your partner(s)?

- 1 - Not at all (1)
- 2 - Slightly (2)
- 3 - Moderately (3)
- 4 - Very (4)

| 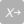 |
| --- |

attchk2 Please select number 2.

- 1 (1)
- 2 (2)
- 3 (3)
- 4 (4)

| 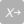 |
| --- |

sens The following items relate to sensation seeking. Please respond to these items using a scale from 1 (not at all like me) to 4 (very much like me).

|  | 1 - Not at all like me (1) | 2 - Not much like me (2) | 3 - Somewhat like me (3) | 4 - Very much like me (4) |
| --- | --- | --- | --- | --- |
| I like wild "unhibited" sexual encounters. (1) |  |  |  |  |
| I have made promises I did not mean to keep to get a person to have sex with me. (2) |  |  |  |  |
| I have felt curious about having anal intercourse without a condom. (3) |  |  |  |  |
| I enjoy the company of "sensual" people. (4) |  |  |  |  |
| I enjoy watching "X-rated" videos. (5) |  |  |  |  |
| I have said things that were not exactly true to get a person to have sex with me. (6) |  |  |  |  |
| I am interested in trying out new sexual experiences. (7) |  |  |  |  |
| I feel like exploring my sexuality. (8) |  |  |  |  |
| I like new and exciting sexual experiences and sensations. (9) |  |  |  |  |

| 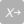 |
| --- |

comp These items relate to compulsive sexual experiences. Please respond to these items using a scale from 1 (not at all like me) to 4 (very much like me).

|  | 1 - Not at all like me (1) | 2 - Not much like me (2) | 3 - Somewhat like me (3) | 4 - Very much like me (4) |
| --- | --- | --- | --- | --- |
| My sexual appetite has gotten in the way of my relationships. (1) |  |  |  |  |
| My sexual thoughts and behaviors are causing problems in my life. (2) |  |  |  |  |
| My desires to have sex have disrupted my daily life. (3) |  |  |  |  |
| I sometimes fail to meet my commitments and responsibilities because of my sexual behaviors. (4) |  |  |  |  |
| I sometimes get so horny I could lose control. (5) |  |  |  |  |
| I find myself thinking about sex while at work. (6) |  |  |  |  |
| I feel that my sexual thoughts and feelings are stronger than I am. (7) |  |  |  |  |
| I have to struggle to control my sexual thoughts and behaviors. (8) |  |  |  |  |
| I think about sex more often than I would like to. (9) |  |  |  |  |
| It has been difficult for me to find sex partners who desire having sex as much as I want to. (10) |  |  |  |  |

| 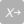 |
| --- |

roug1 People have different ideas about what rough sex means to them. What does it mean to you? Select all that apply.

- None of the following. I don't engage in rough sex. (1)
- Hair pulling (2)
- Being pinned down (3)
- Biting (4)
- Tying up (5)
- Slapping, choking (6)
- Scratching (7)
- Hard thrusting (8)
- Punching (9)
- Spanking (10)
- Throwing someone onto a bed (11)
- Making someone have sex (12)
- Tearing clothes off (13)
- Other (0) __________________________________________________

| Page Break |  |
| --- | --- |

Display This Question:

If Rough Sex (not a scale) != None of the following. I don't engage in rough sex.

| 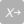 |
| --- |

roug2 How often do you and female sexual partners engage in 'rough sex' together?

- Never (1)
- Rarely (2)
- Sometimes (3)
- Often (4)
- Not Applicable (999)

Display This Question:

If Rough Sex (not a scale) != None of the following. I don't engage in rough sex.

| 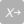 |
| --- |

roug3 How often do you and male sexual partners engage in 'rough sex' together?

- Never (1)
- Rarely (2)
- Sometimes (3)
- Often (4)
- Not Applicable (999)

Display This Question:

If Rough Sex (not a scale) != None of the following. I don't engage in rough sex.

| 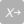 |
| --- |

roug4 In general, how often do you engage in 'rough sex'?

- Never (1)
- Rarely (2)
- Sometimes (3)
- Often (4)
- Not Applicable (999)

Display This Question:

If Rough Sex (not a scale) != None of the following. I don't engage in rough sex.

| 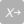 |
| --- |

roug5 option 1 Who usually initiates rough sex?

- 1 - Always you (1)
- 2 - Mostly you (2)
- 3 - Both you and your partner equally (3)
- 4 - Mostly your partner (4)
- 5 - Always your partner (5)

Display This Question:

If Rough Sex (not a scale) != None of the following. I don't engage in rough sex.

| 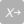 |
| --- |

roug6-32 The following items ask you to what extent you like different forms of rough sex. Please use a scale from 1 (very much) to 4 (not at all) when responding to these questions.

|  | 1 - Very much (1) | 2 - Somewhat (2) | 3 - A little (3) | 4 - Not at all (4) |
| --- | --- | --- | --- | --- |
| To what extent do you like having rough sex? (1) |  |  |  |  |
| To what extent do you like having your hair pulled during sex? (2) |  |  |  |  |
| To what extent do you like being pinned down during sex? (3) |  |  |  |  |
| To what extent do you like being bit during sex? (4) |  |  |  |  |
| To what extent do you like being tied up during sex? (5) |  |  |  |  |
| To what extent do you like being slapped during sex? (6) |  |  |  |  |
| To what extent do you like being choked during sex? (7) |  |  |  |  |
| To what extent do you like being scratched during sex? (8) |  |  |  |  |
| To what extent do you like someone else hard thrusting into you during sex? (9) |  |  |  |  |
| To what extent do you like being punched during sex? (10) |  |  |  |  |
| To what extent do you like being spanked during sex? (11) |  |  |  |  |
| To what extent do you like being thrown onto the bed during sex? (12) |  |  |  |  |
| To what extent do you like someone making you have sex? (13) |  |  |  |  |
| To what extent do you like someone tearing your clothes off during sex? (14) |  |  |  |  |
| To what extent do you like pulling someone else's hair during sex? (15) |  |  |  |  |
| To what extent do you like pinning someone down during sex? (16) |  |  |  |  |
| To what extent do you like biting someone else during sex? (17) |  |  |  |  |
| To what extent do you like tying someone else up during sex? (18) |  |  |  |  |
| To what extent do you like slapping someone else during sex? (19) |  |  |  |  |
| To what extent do you like choking someone else during sex? (20) |  |  |  |  |
| To what extent do you like scratching someone else during sex? (21) |  |  |  |  |
| To what extent do you like hard thrusting into someone else during sex? (22) |  |  |  |  |
| To what extent do you like punching someone else during sex? (23) |  |  |  |  |
| To what extent do you like spanking someone else during sex? (24) |  |  |  |  |
| To what extent do you like throwing someone else onto a bed during sex? (25) |  |  |  |  |
| To what extent do you like making someone else have sex with you? (26) |  |  |  |  |
| To what extent do you like tearing someone else's clothes off during sex? (27) |  |  |  |  |

surveycont. Do you want to continue taking this survey?

- Yes (1)
- I no longer wish to participate (2)

End of Block: Behaviour

Start of Block: Coping with Same-Sex Attraction

| 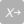 |
| --- |

cop0 Which of the following activities activites have you engaged in to understand, cope with, or change your sexual orientation? Select all that apply.

- Individual effort (e.g., introspection, private study, mental suppression, dating the opposite sex, viewing opposite pornography) (1)
- Personal righteousness (e.g., fasting, prayer, scripture study) (2)
- Psychotherapy (3)
- Psychiatry (medication for depression, anxiety, sleep problems, somatic complaints, and so forth) (4)
- Group therapy (5)
- Group retreats (6)
- Support groups (7)
- Church counseling (e.g., LDS bishops) (8)
- Family therapy (9)

Display This Question:

If Attempts to Cope with Same-Sex Attraction Scale = Individual effort (e.g., introspection, private study, mental suppression, dating the opposite sex, viewing opposite pornography)

cop1a What age were you when you first started engaging in individual efforts (e.g., introspection, private study, mental suppression, dating the opposite sex, viewing opposite-sex pornography) to understand, cope with, or change your sexual orientation?

________________________________________________________________

Display This Question:

If Attempts to Cope with Same-Sex Attraction Scale = Individual effort (e.g., introspection, private study, mental suppression, dating the opposite sex, viewing opposite pornography)

cop1b How many years did you engage in individual efforts (e.g., introspection, private study, mental suppression, dating the opposite sex, viewing opposite-sex pornography) to understand, cope with, or change your sexual orientation?

________________________________________________________________

Display This Question:

If Attempts to Cope with Same-Sex Attraction Scale = Individual effort (e.g., introspection, private study, mental suppression, dating the opposite sex, viewing opposite pornography)

| 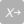 |
| --- |

cop1c How effective do you think it was to engage in individual efforts (e.g., introspection, private study, mental suppression, dating the opposite sex, viewing opposite-sex pornography) to understand, cope with, or change your sexual orientation?

- 1 - Highly effective (1)
- 2 - Moderately effective (2)
- 3 - Not effective (3)
- 4 - Moderately harmful (4)
- 5 - Severely harmful (5)

Display This Question:

If Attempts to Cope with Same-Sex Attraction Scale = Individual effort (e.g., introspection, private study, mental suppression, dating the opposite sex, viewing opposite pornography)

| 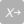 |
| --- |

cop1d What was your original goal for engaging in individual efforts (e.g., introspection, private study, mental suppression, dating the opposite sex, viewing opposite-sex pornography) to understand, cope with, or change your sexual orientation?

- 1 - Desire to change same-sex attraction (1)
- 2 - Desire to accept same-sex attraction (2)

Display This Question:

If Attempts to Cope with Same-Sex Attraction Scale = Individual effort (e.g., introspection, private study, mental suppression, dating the opposite sex, viewing opposite pornography)

cop1e In your own words, please use this space to share about your experiences with individual efforts (e.g., introspection, private study, mental suppression, dating the opposite sex, viewing opposite-sex pornography) to understand, cope with, or change your sexual orientation.

________________________________________________________________

Display This Question:

If Attempts to Cope with Same-Sex Attraction Scale = Personal righteousness (e.g., fasting, prayer, scripture study)

cop2a What age were you when you first started engaging in personal righteousness (e.g., fasting, prayer, scripture study) to understand, cope with, or change your sexual orientation?

________________________________________________________________

Display This Question:

If Attempts to Cope with Same-Sex Attraction Scale = Personal righteousness (e.g., fasting, prayer, scripture study)

cop2b How many years did you engage in personal righteousness (e.g., fasting, prayer, scripture study) to understand, cope with, or change your sexual orientation?

________________________________________________________________

Display This Question:

If Attempts to Cope with Same-Sex Attraction Scale = Personal righteousness (e.g., fasting, prayer, scripture study)

| 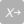 |
| --- |

cop2c How effective do you think it was to engage in personal righteousness (e.g., fasting, prayer, scripture study) to understand, cope with, or change your sexual orientation?

- 1 - Highly effective (1)
- 2 - Moderately effective (2)
- 3 - Not effective (3)
- 4 - Moderately harmful (4)
- 5 - Severely harmful (5)

Display This Question:

If Attempts to Cope with Same-Sex Attraction Scale = Personal righteousness (e.g., fasting, prayer, scripture study)

| 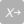 |
| --- |

cop2d What was your original goal for engaging in personal righteousness (e.g., fasting, prayer, scripture study) to understand, cope with, or change your sexual orientation?

- 1 - Desire to change same-sex attraction (1)
- 2 - Desire to accept same-sex attraction (2)

Display This Question:

If Attempts to Cope with Same-Sex Attraction Scale = Personal righteousness (e.g., fasting, prayer, scripture study)

cop2e In your own words, please use this space to share about your experiences with personal righteousness (e.g., fasting, prayer, scripture study) to understand, cope with, or change your sexual orientation.

________________________________________________________________

Display This Question:

If Attempts to Cope with Same-Sex Attraction Scale = Psychotherapy

cop3a What age were you when you first started engaging in psychotherapy to understand, cope with, or change your sexual orientation?

________________________________________________________________

Display This Question:

If Attempts to Cope with Same-Sex Attraction Scale = Psychotherapy

cop3b How many years did you engage in psychotherapy to understand, cope with, or change your sexual orientation?

________________________________________________________________

Display This Question:

If Attempts to Cope with Same-Sex Attraction Scale = Psychotherapy

| 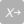 |
| --- |

cop3c How effective do you think it was to engage in psychotherapy to understand, cope with, or change your sexual orientation?

- 1 - Highly effective (1)
- 2 - Moderately effective (2)
- 3 - Not effective (3)
- 4 - Moderately harmful (4)
- 5 - Severely harmful (5)

Display This Question:

If Attempts to Cope with Same-Sex Attraction Scale = Psychotherapy

| 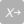 |
| --- |

cop3d What was your original goal for engaging in psychotherapy to understand, cope with, or change your sexual orientation?

- 1 - Desire to change same-sex attraction (1)
- 2 - Desire to accept same-sex attraction (2)

Display This Question:

If Attempts to Cope with Same-Sex Attraction Scale = Psychotherapy

cop3e In your own words, please use this space to share about your experiences with psychotherapy to understand, cope with, or change your sexual orientation.

________________________________________________________________

Display This Question:

If Attempts to Cope with Same-Sex Attraction Scale = Psychiatry (medication for depression, anxiety, sleep problems, somatic complaints, and so forth)

cop4a What age were you when you first started engaging in psychiatry (medication for depression, anxiety, sleep problems, somatic complaints, and so forth) to understand, cope with, or change your sexual orientation?

________________________________________________________________

Display This Question:

If Attempts to Cope with Same-Sex Attraction Scale = Psychiatry (medication for depression, anxiety, sleep problems, somatic complaints, and so forth)

cop4b How many years did you engage in psychiatry (medication for depression, anxiety, sleep problems, somatic complaints, and so forth) to understand, cope with, or change your sexual orientation?

________________________________________________________________

Display This Question:

If Attempts to Cope with Same-Sex Attraction Scale = Psychiatry (medication for depression, anxiety, sleep problems, somatic complaints, and so forth)

| 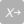 |
| --- |

cop4c How effective do you think it was to engage in psychiatry (medication for depression, anxiety, sleep problems, somatic complaints, and so forth) to understand, cope with, or change your sexual orientation?

- 1 - Highly effective (1)
- 2 - Moderately effective (2)
- 3 - Not effective (3)
- 4 - Moderately harmful (4)
- 5 - Severely harmful (5)

Display This Question:

If Attempts to Cope with Same-Sex Attraction Scale = Psychiatry (medication for depression, anxiety, sleep problems, somatic complaints, and so forth)

| 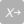 |
| --- |

cop4d What was your original goal for engaging in psychiatry (medication for depression, anxiety, sleep problems, somatic complaints, and so forth) to understand, cope with, or change your sexual orientation?

- 1 - Desire to change same-sex attraction (1)
- 2 - Desire to accept same-sex attraction (2)

Display This Question:

If Attempts to Cope with Same-Sex Attraction Scale = Psychiatry (medication for depression, anxiety, sleep problems, somatic complaints, and so forth)

cop4e In your own words, please use this space to share about your experiences with psychiatry (medication for depression, anxiety, sleep problems, somatic complaints, and so forth) to understand, cope with, or change your sexual orientation.

________________________________________________________________

Display This Question:

If Attempts to Cope with Same-Sex Attraction Scale = Group therapy

cop5a What age were you when you first started engaging in group therapy to understand, cope with, or change your sexual orientation?

________________________________________________________________

Display This Question:

If Attempts to Cope with Same-Sex Attraction Scale = Group therapy

cop5b How many years did you engage in group therapy to understand, cope with, or change your sexual orientation?

________________________________________________________________

Display This Question:

If Attempts to Cope with Same-Sex Attraction Scale = Group therapy

| 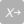 |
| --- |

cop5c How effective do you think it was to engage in group therapy to understand, cope with, or change your sexual orientation?

- 1 - Highly effective (1)
- 2 - Moderately effective (2)
- 3 - Not effective (3)
- 4 - Moderately harmful (4)
- 5 - Severely harmful (5)

Display This Question:

If Attempts to Cope with Same-Sex Attraction Scale = Group therapy

| 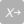 |
| --- |

cop5d What was your original goal for engaging in group therapy to understand, cope with, or change your sexual orientation?

- 1 - Desire to change same-sex attraction (1)
- 2 - Desire to accept same-sex attraction (2)

Display This Question:

If Attempts to Cope with Same-Sex Attraction Scale = Group therapy

cop5e In your own words, please use this space to share about your experiences with group therapy to understand, cope with, or change your sexual orientation.

________________________________________________________________

Display This Question:

If Attempts to Cope with Same-Sex Attraction Scale = Group retreats

cop6a What age were you when you first started engaging in group retreats to understand, cope with, or change your sexual orientation?

________________________________________________________________

Display This Question:

If Attempts to Cope with Same-Sex Attraction Scale = Group retreats

cop6b How many years did you engage in group retreats to understand, cope with, or change your sexual orientation?

________________________________________________________________

Display This Question:

If Attempts to Cope with Same-Sex Attraction Scale = Group retreats

| 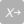 |
| --- |

cop6c How effective do you think it was to engage in group retreats to understand, cope with, or change your sexual orientation?

- 1 - Highly effective (1)
- 2 - Moderately effective (2)
- 3 - Not effective (3)
- 4 - Moderately harmful (4)
- 5 - Severely harmful (5)

Display This Question:

If Attempts to Cope with Same-Sex Attraction Scale = Group retreats

| 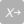 |
| --- |

cop6d What was your original goal for engaging in group retreats to understand, cope with, or change your sexual orientation?

- 1 - Desire to change same-sex attraction (1)
- 2 - Desire to accept same-sex attraction (2)

Display This Question:

If Attempts to Cope with Same-Sex Attraction Scale = Group retreats

cop6e In your own words, please use this space to share about your experiences with group retreats to understand, cope with, or change your sexual orientation.

________________________________________________________________

Display This Question:

If Attempts to Cope with Same-Sex Attraction Scale = Support groups

cop7a What age were you when you first started engaging in support groups to understand, cope with, or change your sexual orientation?

________________________________________________________________

Display This Question:

If Attempts to Cope with Same-Sex Attraction Scale = Support groups

cop7b How many years did you engage in support groups to understand, cope with, or change your sexual orientation?

________________________________________________________________

Display This Question:

If Attempts to Cope with Same-Sex Attraction Scale = Support groups

| 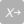 |
| --- |

cop7c How effective do you think it was to engage in support groups to understand, cope with, or change your sexual orientation?

- 1 - Highly effective (1)
- 2 - Moderately effective (2)
- 3 - Not effective (3)
- 4 - Moderately harmful (4)
- 5 - Severely harmful (5)

Display This Question:

If Attempts to Cope with Same-Sex Attraction Scale = Support groups

| 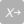 |
| --- |

cop7d What was your original goal for engaging in support groups to understand, cope with, or change your sexual orientation?

- 1 - Desire to change same-sex attraction (1)
- 2 - Desire to accept same-sex attraction (2)

Display This Question:

If Attempts to Cope with Same-Sex Attraction Scale = Support groups

cop7e In your own words, please use this space to share about your experiences with support groups to understand, cope with, or change your sexual orientation.

________________________________________________________________

Display This Question:

If Attempts to Cope with Same-Sex Attraction Scale = Church counseling (e.g., LDS bishops)

cop8a What age were you when you first started engaging in church counseling (e.g., LDS bishops) to understand, cope with, or change your sexual orientation?

________________________________________________________________

Display This Question:

If Attempts to Cope with Same-Sex Attraction Scale = Church counseling (e.g., LDS bishops)

cop8b How many years did you engage in church counseling (e.g., LDS bishops) to understand, cope with, or change your sexual orientation?

________________________________________________________________

Display This Question:

If Attempts to Cope with Same-Sex Attraction Scale = Church counseling (e.g., LDS bishops)

| 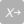 |
| --- |

cop8c How effective do you think it was to engage in church counseling (e.g., LDS bishops) to understand, cope with, or change your sexual orientation?

- 1 - Highly effective (1)
- 2 - Moderately effective (2)
- 3 - Not effective (3)
- 4 - Moderately harmful (4)
- 5 - Severely harmful (5)

Display This Question:

If Attempts to Cope with Same-Sex Attraction Scale = Church counseling (e.g., LDS bishops)

| 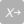 |
| --- |

cop8d How effective do you think it was to engage in church counseling (e.g., LDS bishops) to understand, cope with, or change your sexual orientation?

- 1 - Desire to change same-sex attraction (1)
- 2 - Desire to accept same-sex attraction (2)

Display This Question:

If Attempts to Cope with Same-Sex Attraction Scale = Church counseling (e.g., LDS bishops)

cop8e In your own words, please use this space to share about your experiences with church counseling (e.g., LDS bishops) to understand, cope with, or change your sexual orientation.

________________________________________________________________

Display This Question:

If Attempts to Cope with Same-Sex Attraction Scale = Family therapy

cop9a What age were you when you first started engaged in family therapy to understand, cope with, or change your sexual orientation?

________________________________________________________________

Display This Question:

If Attempts to Cope with Same-Sex Attraction Scale = Family therapy

cop9b How many years did you engage in family therapy to understand, cope with, or change your sexual orientation?

________________________________________________________________

Display This Question:

If Attempts to Cope with Same-Sex Attraction Scale = Family therapy

| 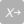 |
| --- |

cop9c How effective do you think it was to engage in family therapy to understand, cope with, or change your sexual orientation?

- 1 - Highly effective (1)
- 2 - Moderately effective (2)
- 3 - Not effective (3)
- 4 - Moderately harmful (4)
- 5 - Severely harmful (5)

Display This Question:

If Attempts to Cope with Same-Sex Attraction Scale = Family therapy

| 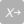 |
| --- |

cop9d What was your original goal for engaging in family therapy to understand, cope with, or change your sexual orientation?

- 1 - Desire to change same-sex attraction (1)
- 2 - Desire to accept same-sex attraction (2)

Display This Question:

If Attempts to Cope with Same-Sex Attraction Scale = Family therapy

cop9e In your own words, please use this space to share about your experiences with family therapy to understand, cope with, or change your sexual orientation.

________________________________________________________________

| 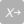 |
| --- |

surveycont. Do you want to continue taking this survey?

- Yes (1)
- I no longer wish to participate (2)

End of Block: Coping with Same-Sex Attraction

Start of Block: Relationship Negotiation and Communication

| 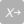 |
| --- |

scrp The following items relate to your opinions on how men and women should act. Please use the scale from 1 (strongly disagree) to 6 (strongly agree) when responding to each item.

|  | 1 - Strongly disagree (1) | 2 - Disagree (2) | 3 - Somewhat disagree (3) | 4 - Somewhat agree (4) | 5 - Agree (5) | 6 - Strongly agree (6) |
| --- | --- | --- | --- | --- | --- | --- |
| The best way for a girl to attract a boyfriend is to use her body and looks. (1) |  |  |  |  |  |  |
| There is nothing wrong with men being primarily interested in a woman’s body. (2) |  |  |  |  |  |  |
| No matter what she says, a girl isn’t really happy unless she’s in a relationship. (3) |  |  |  |  |  |  |
| Girls should do whatever they need to (e.g., use make-up, buy attractive clothes, and work out) to look good enough to attract a date/partner. (4) |  |  |  |  |  |  |
| Sometimes girls have to do things they don’t want to do to keep their boyfriend happy. (5) |  |  |  |  |  |  |
| A woman should be willing to make personal sacrifices in order to satisfy her partner. (6) |  |  |  |  |  |  |
| Guys like to play the field and shouldn’t be expected to stay with one partner for too long. (7) |  |  |  |  |  |  |
| Women are attracted most to a man with a lot of money. (8) |  |  |  |  |  |  |
| A man should always protect and defend his woman. (9) |  |  |  |  |  |  |
| Men should be the ones to ask women out and initiate physical contact. (10) |  |  |  |  |  |  |
| A woman wants a man because she wants someone to protect her. (11) |  |  |  |  |  |  |
| Women like to admire men’s bodies and are attracted most to men who are muscular and handsome. (12) |  |  |  |  |  |  |
| Being with an attractive partner gives a guy prestige. (13) |  |  |  |  |  |  |
| Guys who are able to date a lot of people (players) are considered cool. (14) |  |  |  |  |  |  |
| In the dating game, guys frequently compete with each other for partners, and girls try to lure or catch partners. (15) |  |  |  |  |  |  |
| It’s only natural for a guy to make advances on someone he finds attractive. (16) |  |  |  |  |  |  |
| It is natural for a guy to want to admire or check out other people, even if he is dating someone. (17) |  |  |  |  |  |  |
| Guys are always ready for sex. (18) |  |  |  |  |  |  |
| Most guys don’t want to be ‘‘just friends’’ with a girl. (19) |  |  |  |  |  |  |
| Guys are more interested in physical relationships and girls are more interested in emotional relationships. (20) |  |  |  |  |  |  |
| It is up to women to keep things from moving too fast sexually. (21) |  |  |  |  |  |  |
| Women with a lot of ‘‘experience’’ should expect a bad reputation. (22) |  |  |  |  |  |  |

Display This Question:

If Have you ever been sexually active with a woman? Sexual activity is broad and includes not only s... = Yes

| 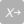 |
| --- |

sec1a-3a The following questions ask about your thoughts and feelings concerning sexual activities with a partner and your sexual experiences. You are asked to rate each item on the scales provided. Please check off one box per item to indicate your response. The sexual partner described in these questions below may or may not be a committed relationship partner.

|  | 1 - Very difficult (1) | 2 - Moderately difficult (2) | 3 - Slightly difficult (3) | 4 - Neither easy nor difficult (4) | 5 - Slightly easy (5) | 6 - Moderately easy (6) | 7 - Very easy (7) |
| --- | --- | --- | --- | --- | --- | --- | --- |
| Telling a female sexual partner what to do to stimulate me during intercourse would be: (1) |  |  |  |  |  |  |  |
| Showing a female sexual partner what to do to stimulate me during intercourse would be: (2) |  |  |  |  |  |  |  |
| Asking a female sexual partner to stimulate me to orgasm (i.e., by massaging my genitals) when I have intercourse with them would be: (3) |  |  |  |  |  |  |  |

Display This Question:

If Have you ever been sexually active with a woman? Sexual activity is broad and includes not only s... = Yes

| 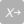 |
| --- |

sec4a-6a The following questions ask about your thoughts and feelings concerning sexual activities with a partner and your sexual experiences. You are asked to rate each item on the scales provided. Please check off one box per item to indicate your response. The sexual partner described in these questions below may or may not be a committed relationship partner.

|  | 0 - I have never had sex with a female sexual partner (0) | 1 - 0% of the time (1) | 2 - 1-25% of the time (2) | 3 - 26-50% of the time (3) | 4 - 51-75% of the time (4) | 5 - 76-99% of the time (5) | 6 - 100% of the time (6) |
| --- | --- | --- | --- | --- | --- | --- | --- |
| When having sex with a female sexual partner, how often do you tell your sexual partners what feels good? (1) |  |  |  |  |  |  |  |
| When having sex with a female sexual partner, how often do you show your sexual partners what feels good? (2) |  |  |  |  |  |  |  |
| When having sex with a female sexual partner, how often do you ask your sexual partners to stimulate your genitals to orgasm? (3) |  |  |  |  |  |  |  |

Display This Question:

If Have you ever been sexually active with a woman? Sexual activity is broad and includes not only s... = Yes

| 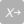 |
| --- |

sec7a-9a The following questions ask about your thoughts and feelings concerning sexual activities with a partner and your sexual experiences. You are asked to rate each item on the scales provided. Please check off one box per item to indicate your response. The sexual partner described in these questions below may or may not be a committed relationship partner.

|  | 1 - Very difficult (1) | 2 - Moderately difficult (2) | 3 - Slightly difficult (3) | 4 - Neither easy nor difficult (4) | 5 - Slightly easy (5) | 6 - Moderately easy (6) | 7 - Very easy (7) |
| --- | --- | --- | --- | --- | --- | --- | --- |
| Talking to a female sexual partner about what they would like done to stimulate them during intercourse would be: (1) |  |  |  |  |  |  |  |
| Having a female sexual partner show me what to do to stimulate them during intercourse would be: (2) |  |  |  |  |  |  |  |
| If, when I had intercourse with a female sexual partner, they asked me to stimulate them to orgasm in a way that is different from the way I would normally prefer, it would be: (3) |  |  |  |  |  |  |  |

Display This Question:

If Have you ever been sexually active with a woman? Sexual activity is broad and includes not only s... = Yes

| 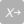 |
| --- |

sec10a-12a The following questions ask about your thoughts and feelings concerning sexual activities with a partner and your sexual experiences. You are asked to rate each item on the scales provided. Please check off one box per item to indicate your response. The sexual partner described in these questions below may or may not be a committed relationship partner.

|  | 0 - I have never had sex with a female sexual partner (0) | 1 - 0% of the time (1) | 2 - 1-25% of the time (2) | 3 - 26-50% of the time (3) | 4 - 51-75% of the time (4) | 5 - 76-99% of the time (5) | 6 - 100% of the time (6) |
| --- | --- | --- | --- | --- | --- | --- | --- |
| When having sex with a female sexual partner, how often do you tell your sexual partners what feels good? (1) |  |  |  |  |  |  |  |
| When having sex with a female sexual partner, how often do you show your sexual partners what feels good? (2) |  |  |  |  |  |  |  |
| When having sex with a female sexual partner, how often do you ask your sexual partners to stimulate your genitals to orgasm? (3) |  |  |  |  |  |  |  |

Display This Question:

If sexman = Yes

| 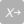 |
| --- |

sec1b-3b The following questions ask about your thoughts and feelings concerning sexual activities with a partner and your sexual experiences. You are asked to rate each item on the scales provided. Please check off one box per item to indicate your response. The sexual partner described in these questions below may or may not be a committed relationship partner.

|  | 1 - Very difficult (1) | 2 - Moderately difficult (2) | 3 - Slightly difficult (3) | 4 - Neither easy nor difficult (4) | 5 - Slightly easy (5) | 6 - Moderately easy (6) | 7 - Very easy (7) |
| --- | --- | --- | --- | --- | --- | --- | --- |
| Telling a male sexual partner what to do to stimulate me during intercourse would be: (1) |  |  |  |  |  |  |  |
| Showing a male sexual partner what to do to stimulate me during intercourse would be: (2) |  |  |  |  |  |  |  |
| Asking a male sexual partner to stimulate me to orgasm (i.e., by massaging my genitals) when I have intercourse with them would be: (3) |  |  |  |  |  |  |  |

Display This Question:

If sexman = Yes

| 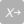 |
| --- |

sec4b-6b The following questions ask about your thoughts and feelings concerning sexual activities with a partner and your sexual experiences. You are asked to rate each item on the scales provided. Please check off one box per item to indicate your response. The sexual partner described in these questions below may or may not be a committed relationship partner.

|  | 0 - I have never had sex with a male sexual partner (0) | 1 - 0% of the time (1) | 2 - 1-25% of the time (2) | 3 - 26-50% of the time (3) | 4 - 51-75% of the time (4) | 5 - 76-99% of the time (5) | 6 - 100% of the time (6) |
| --- | --- | --- | --- | --- | --- | --- | --- |
| When having sex with a male sexual partner, how often do you tell your sexual partners what feels good? (1) |  |  |  |  |  |  |  |
| When having sex with a male sexual partner, how often do you show your sexual partners what feels good? (2) |  |  |  |  |  |  |  |
| When having sex with a male sexual partner, how often do you ask your sexual partners to stimulate your genitals to orgasm? (3) |  |  |  |  |  |  |  |

Display This Question:

If sexman = Yes

| 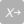 |
| --- |

sec7b-9b The following questions ask about your thoughts and feelings concerning sexual activities with a partner and your sexual experiences. You are asked to rate each item on the scales provided. Please check off one box per item to indicate your response. The sexual partner described in these questions below may or may not be a committed relationship partner.

|  | 1 - Very difficult (1) | 2 - Moderately difficult (2) | 3 - Slightly difficult (3) | 4 - Neither easy nor difficult (4) | 5 - Slightly easy (5) | 6 - Moderately easy (6) | 7 - Very easy (7) |
| --- | --- | --- | --- | --- | --- | --- | --- |
| Talking to a male sexual partner about what they would like done to stimulate them during intercourse would be: (1) |  |  |  |  |  |  |  |
| Having a male sexual partner show me what to do to stimulate them during intercourse would be: (2) |  |  |  |  |  |  |  |
| If, when I had intercourse with a male sexual partner, they asked me to stimulate them to orgasm in a way that is different from the way I would normally prefer, it would be: (3) |  |  |  |  |  |  |  |

Display This Question:

If sexman = Yes

| 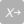 |
| --- |

sec10b-12b The following questions ask about your thoughts and feelings concerning sexual activities with a partner and your sexual experiences. You are asked to rate each item on the scales provided. Please check off one box per item to indicate your response. The sexual partner described in these questions below may or may not be a committed relationship partner.

|  | 0 - I have never had sex with a male sexual partner (0) | 1 - 0% of the time (1) | 2 - 1-25% of the time (2) | 3 - 26-50% of the time (3) | 4 - 51-75% of the time (4) | 5 - 76-99% of the time (5) | 6 - 100% of the time (6) |
| --- | --- | --- | --- | --- | --- | --- | --- |
| When having sex with a male sexual partner, how often do you tell your sexual partners what feels good? (1) |  |  |  |  |  |  |  |
| When having sex with a male sexual partner, how often do you show your sexual partners what feels good? (2) |  |  |  |  |  |  |  |
| When having sex with a male sexual partner, how often do you ask your sexual partners to stimulate your genitals to orgasm? (3) |  |  |  |  |  |  |  |

| 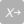 |
| --- |

surveycont. Do you want to continue taking this survey?

- Yes (1)
- I no longer wish to participate (2)

End of Block: Relationship Negotiation and Communication

Start of Block: Technology Usage

| 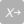 |
| --- |

iiaf On a scale of 1 (never) to 4 (a lot), please indicate how frequently you have accidentally or unexpectedly learned information in the following ways.

|  | 1 - Never (1) | 2 - Rarely (2) | 3 - Sometimes (3) | 4 - A lot (4) |
| --- | --- | --- | --- | --- |
| I accidently find information about HIV/AIDS while I look for information about other topics. (1) |  |  |  |  |
| I learn unexpected things about HIV/AIDS when I watch television or read the newspaper. (2) |  |  |  |  |
| I learn unexpected things about HIV/AIDS when I talk to other people. (3) |  |  |  |  |

| 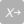 |
| --- |

inuse The following questions ask about how often you use the internet in different situations. Please use the scale from 0 (never) to 4 (several times a day).

|  | 0 - Never (0) | 1 - Less often than every few weeks (1) | 2 - Every few weeks (2) | 3 - Several times a week (3) | 4 - Several times a day (4) |
| --- | --- | --- | --- | --- | --- |
| How often do you use the Internet at home? (1) |  |  |  |  |  |
| How often do you use the Internet at school? (2) |  |  |  |  |  |
| How often do you use the Internet at work? (3) |  |  |  |  |  |
| How often do you use the Internet at public library or community center? (4) |  |  |  |  |  |
| How often do you use the Internet on a mobile device? (5) |  |  |  |  |  |
| How often do you use the Internet in another location? (6) |  |  |  |  |  |

| 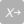 |
| --- |

onli1-3 The following items ask about how often you typically access different online websites.

|  | 1 - Less than once (1) | 2 - Once (2) | 3 - 2-5 times (3) | 4 - 6-10 times (4) | 5 - 11-15 times (5) | 6 - More than 15 times (6) | 7 - Almost constantly (7) |
| --- | --- | --- | --- | --- | --- | --- | --- |
| On a typical DAY, how often do you go online in general? (1) |  |  |  |  |  |  |  |
| On a typical DAY, how often do you access social media (including using social media apps or in a web browser)? Social media includes websites like Facebook, Instagram, Twitter, Reddit, and Tumblr. (2) |  |  |  |  |  |  |  |
| On a typical DAY, how often do you access messaging apps or platforms? Messaging includes websites and platforms like text messages/SMS, iMessage, WhatsApp, Facebook Messenger, Kik, and Snapchat? (3) |  |  |  |  |  |  |  |

| 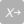 |
| --- |

onli4 Which types of apps do you use? Please select all that apply.

- Dating (e.g., Hinge, Plenty of Fish) (1)
- Hook-Up Apps (e.g., Grindr, Tinder) (2)
- Media Sharing (e.g., Instagram, Snapchat, TikTok, YouTube) (3)
- Messaging (e.g., WhatsApp, WeChat) (4)
- Social Networking (e.g., Facebook, X) (5)

| 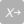 |
| --- |

onli5 Which of these devices do you use to go online at least once a week? Select all that apply.

- Cell/mobile phone (1)
- Tablet (e.g., iPad) (2)
- Laptop (3)
- Desktop PC (4)
- Portable media player (e.g., iPod) (5)
- SmartTV (6)
- Wearable device (e.g., smartwatch) (7)
- Handheld gaming device (e.g., PS VITA, Nintendo Switch) (8)
- Gaming console (e.g., PS4, Xbox One) (9)
- Other: (0) __________________________________________________

| 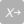 |
| --- |

onli6 Do you use a "Screen Time" or "Digital Wellbeing" app or feature to track your screen use on the device you use the most?

- 1 - Yes (1)
- 2 - No (2)
- 3 - Unsure (3)

| Page Break |  |
| --- | --- |

Display This Question:

If Social Media Use (not a scale) = 1 - Yes

onli7a If yes, please share your usage. Below we provide examples of how to do this on Apple's Screen Time and Google's Digital Wellbeing. However, many other applications exist for different operating systems.

Instructions for iPhone/iPad:
1. Go to Settings > Screen Time App
2. Select Last 7 Days
3. Find the number inside the red box
4. Type in the number below
5. For Duration, select Last Seven Days

Instructions for Android:
1. Go to Settings > Digital Well-Being App
2. Find the number inside the red box
3. Type in the number below
4. For Duration, select Today

________________________________________________________________

Display This Question:

If Social Media Use (not a scale) = 1 - Yes

| 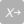 |
| --- |

onli7b Select duration:

- 1 - Today (1)
- 2 - Yesterday (2)
- 3 - Last seven days (3)
- 4 - Other amount of time (4) __________________________________________________

Display This Question:

If Social Media Use (not a scale) = 1 - Yes

onli7c What does your device say about your 5 most used (i.e., time spent) apps? Please list in order from most to least used.

________________________________________________________________

Display This Question:

If Social Media Use (not a scale) = 1 - Yes

onli7d Please share a screenshot of your time tracking screen.

| 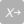 |
| --- |

onli8 Please indicate if you go online (including using social networking websites and internet-enabled apps and platforms) in the following situations: (Select all that apply)

- At school (1)
- At work (2)
- While commuting (e.g., bus, train) (3)
- During meals (4)
- While studying (5)
- In social situations with friends (6)
- In social situations with family (7)
- While playing sports/at the gym (8)
- While watching videos (e.g., television, YouTube) (9)
- Before I go to bed (10)
- When I wake up during the night (11)
- When I wake up in the morning (12)
- Other (0) __________________________________________________

onli9 What types of online communities or groups do you like to spend time in on the Internet?

________________________________________________________________

onli10 Is there anything else you think we should know about how you use technology or access the Internet?

________________________________________________________________

| 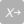 |
| --- |

attchk3 Please select option 3.

- 1 (1)
- 2 (2)
- 3 (3)
- 4 (4)

| 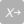 |
| --- |

idat The following items ask how likely or unlikely it is that you will use dating apps in the coming week. Please respond to these items using a scale from 1 (very unlikely) to 7 (very likely).

|  | 1 - Very unlikely (1) | 2 - Unlikely (2) | 3 - Somewhat unlikely (3) | 4 - Neither unlikely or likely (4) | 5 - Somewhat likely (5) | 6 - Likely (6) | 7 - Very likely (7) |
| --- | --- | --- | --- | --- | --- | --- | --- |
| In the coming week, how likely or unlikely I will use a dating app to look for a casual sexual partner? (1) |  |  |  |  |  |  |  |
| In the coming week, how likely or unlikely I will use a dating app to talk/respond to someone who is looking for casual sex? (2) |  |  |  |  |  |  |  |
| In the coming week, how likely or unlikely I will use a dating app to arrange a time and a place to hook up? (3) |  |  |  |  |  |  |  |
| In the coming week, how likely or unlikely I will use a dating app to share a sexy photo of yourself to others? (4) |  |  |  |  |  |  |  |
| In the coming week, how likely or unlikely I will use a dating app to tell others about your interests and hobbies? (5) |  |  |  |  |  |  |  |
| In the coming week, how likely or unlikely I will use a dating app to look for a romantic partner? (6) |  |  |  |  |  |  |  |
| In the coming week, how likely or unlikely I will use a dating app to talk/respond to someone whom you might be romantically interested in? (7) |  |  |  |  |  |  |  |
| In the coming week, how likely or unlikely I will use a dating app to share with others your work/school life? (8) |  |  |  |  |  |  |  |

| 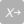 |
| --- |

adat These items ask about your attitudes toward using dating apps. Please use the scale from 1 (strongly disagree) to 7 (strongly agree).

|  | 1 - Strongly disagree (1) | 2 - Disagree (2) | 3 - Somewhat disagree (3) | 4 - Neither agree nor disagree (4) | 5 - Somewhat agree (5) | 6 - Agree (6) | 7 - Strongly agree (7) |
| --- | --- | --- | --- | --- | --- | --- | --- |
| I think there is more good than bad in using dating apps to look for romantic partners. (1) |  |  |  |  |  |  |  |
| Using dating apps to look for romantic partners is better than meeting potential romantic partners on a face-to-face occasion. (2) |  |  |  |  |  |  |  |
| Using dating apps is the best way to look for a romantic partner these days. (3) |  |  |  |  |  |  |  |
| I think there is more good than bad in using dating apps to look for casual sexual partners. (4) |  |  |  |  |  |  |  |
| Using dating apps to look for casual sexual partners is better than meeting potential casual sexual partners on a face-to-face occasion. (5) |  |  |  |  |  |  |  |
| Using dating apps is the best way to look for a casual sexual partner these days. (6) |  |  |  |  |  |  |  |

| 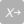 |
| --- |

priv1 On dating/hook up apps, do you have a face picture on your profile?

- Yes (1)
- No (2)

| 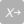 |
| --- |

priv2 On dating/hook up apps, do you send other people a clear picture of your face upon request?

- Yes (1)
- No (2)

| 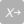 |
| --- |

priv3 On dating/hook up apps, do you use your real name as your username?

- Yes (1)
- No (2)

| 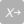 |
| --- |

priv4 On dating/hook up apps, do you tell other people your real name during conversations?

- Yes (1)
- No (2)

| 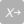 |
| --- |

priv5 On dating/hook up apps, do you state your HIV status on your profile?

- Yes (1)
- No (2)

| 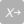 |
| --- |

grind1 How often do you log on to Grindr?

- I do not use Grindr (999)
- 1 - 5 or more times a day (1)
- 2 - More than once a day but less than 5 times per day (2)
- 3 - Once a day (3)
- 4 - A few days a week (4)
- 5 - About once a week (5)
- 6 - Less than once a week (6)

| Page Break |  |
| --- | --- |

Display This Question:

If Grindr Use != I do not use Grindr

| 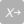 |
| --- |

grind2 When did you start using Grindr?

- 1 - Less than one month ago (1)
- 2 - More than 1 month, but less than 6 months ago (2)
- 3 - More than 6 months, but less than 1 year ago (3)
- 4 - More than 1 year ago (4)

Display This Question:

If Grindr Use != I do not use Grindr

|  |
| --- |

grind3 Is your face visible on your Grindr profile?

- 1 - Yes (1)
- 2 - No (2)

Display This Question:

If Grindr Use != I do not use Grindr

|  |
| --- |

grind4 In your photo, do you show any of the following naked body parts? Select all that apply.

- Chest (1)
- Abs (2)
- Arms/biceps (3)
- Back/shoulders (4)
- Legs (5)
- None (999)

Display This Question:

If Grindr Use != I do not use Grindr

|  |
| --- |

grind5 When you use Grindr, what time of day is it usually?

- Morning (1)
- Afternoon (2)
- Evening (3)
- Night (before midnight) (4)
- Late night (after midnight) (5)

Display This Question:

If Grindr Use != I do not use Grindr

|  |
| --- |

grind6 Do you primarily use Grindr on:

- 1 - Weekends (1)
- 2 - Weekdays (2)
- 3 - Both (3)

Display This Question:

If Grindr Use != I do not use Grindr

|  |
| --- |

grind7 The last time you used Grindr, was it during or immediately after you had been drinking or using drugs?

- 1 - Yes (1)
- 2 - No (2)
- 3 - I don't know (3)

Display This Question:

If Grindr Use != I do not use Grindr

|  |
| --- |

grind8 What are the reasons you use Grindr?

- To make new friends (1)
- To meet people to have sex/hook up with (2)
- To find someone to date (3)
- To "kill" time (4)
- To connect to the gay community (5)
- To find people to drink/use drugs with (6)
- To communicate with in-person friends (7)
- To reconnect with people from the past (8)

Display This Question:

If Grindr Use != I do not use Grindr

|  |
| --- |

grind9 What is your number one reason for using Grindr?

- To make new friends (1)
- To meet people to have sex/hook up with (2)
- To find someone to date (3)
- To "kill" time (4)
- To connect to the gay community (5)
- To find people to drink/use drugs with (6)
- To communicate with in-person friends (7)
- To reconnect with people from the past (8)

Display This Question:

If Grindr Use != I do not use Grindr

|  |
| --- |

grind0 Have you ever had sex with a partner you met on Grindr?

- 1 - Yes (1)
- 2 - No (2)

|  |
| --- |

surveycont. Do you want to continue taking this survey?

- Yes (1)
- I no longer wish to participate (2)

End of Block: Technology Usage

Start of Block: Health & Well-Being

|  |
| --- |

prep0 Do you currently take the medication commonly known as PrEP (pre-exposure prophylaxis)? Common names include Truvada and Descovy.

- 0 - No (1)
- 1 - Yes (2)
- 2 - I don't know (3)

|  |
| --- |

kpre For each of the following questions, please choose True (T), False (F), or Don’t Know (DK). If you do not know, please do not guess; instead, please choose Don’t Know.

|  | 1 - True (1) | 2 - False (2) | 3 - Don't know (3) |
| --- | --- | --- | --- |
| PrEP is a daily pill you can take to reduce your risk of becoming infected with HIV. (1) |  |  |  |
| You should not use PrEP if you don’t know your HIV status. (2) |  |  |  |
| If you do not take PrEP consistently, there may not be enough medicine in your bloodstream to block the HIV virus. (3) |  |  |  |
| PrEP can be used to prevent STIs like gonorrhea, chlamydia, syphilis, herpes, and HPV. (4) |  |  |  |
| Many insurance plans will cover PrEP. (5) |  |  |  |
| If you start taking PrEP, you will have to take it for the rest of your life. (6) |  |  |  |
| PrEP can be taken by people who already have HIV. (7) |  |  |  |
| There are ways to get PrEP even if you do not have insurance. (8) |  |  |  |
| PrEP is available over-the-counter. (9) |  |  |  |
| You must take an HIV test every 3 months while taking PrEP. (10) |  |  |  |
| There are many serious side effects of taking PrEP. (11) |  |  |  |
| The PrEP pill contains two medicines that are also used to treat HIV. (12) |  |  |  |
| Daily PrEP use can lower the risk of getting HIV from sex by more than 90%. (13) |  |  |  |

|  |
| --- |

aprep Do you agree or disagree with the following statements?

|  | 1 - Strongly disagree (1) | 2 - Disagree (2) | 3 - Neutral (3) | 4 - Agree (4) | 5 - Strongly agree (5) |
| --- | --- | --- | --- | --- | --- |
| PrEP is effective at preventing HIV. (1) |  |  |  |  |  |
| People who take PrEP are responsible. (2) |  |  |  |  |  |
| Taking PrEP is safe. (3) |  |  |  |  |  |
| It would be no trouble to take PrEP every day. (4) |  |  |  |  |  |
| The government makes certain that drugs like PrEP are safe. (5) |  |  |  |  |  |

|  |
| --- |

PrEP/PEPStatements Do you agree or disagree with the following statements?

|  | 1 - Strongly disagree (1) | 2 - Disagree (2) | 3 - Neutral (3) | 4 - Agree (4) | 5 - Strongly agree (5) |
| --- | --- | --- | --- | --- | --- |
| People who take PrEP are promiscuous. (1) |  |  |  |  |  |
| Getting a PrEP prescription from a doctor would be embarrassing. (2) |  |  |  |  |  |
| If I were to take PrEP, I would be concerned if my friends found out I was taking it. (3) |  |  |  |  |  |
| If I were to take PrEP, I would be concerned if my family found out I was taking it. (4) |  |  |  |  |  |
| If I were to take PrEP, I would be concerned if my sexual partner(s) found out I was taking it. (5) |  |  |  |  |  |

|  |
| --- |

lifesat Below are five statements that you may agree or disagree with. Using the 1 - 5 scale below, indicate your agreement with each item by placing the appropriate number on the line preceding that item. Please be open and honest in your responding.

|  | 1 - Strongly disagree (1) | 2 - Disagree (2) | 3 - Neutral (3) | 4 - Agree (4) | 5 - Strongly agree (5) |
| --- | --- | --- | --- | --- | --- |
| In most ways my life is close to ideal. (1) |  |  |  |  |  |
| The conditions of my life are excellent. (2) |  |  |  |  |  |
| I am satisfied with my life. (3) |  |  |  |  |  |
| So far I have gotten the important things I want in life. (4) |  |  |  |  |  |
| If I could live with my life over, I would change almost nothing. (5) |  |  |  |  |  |

|  |
| --- |

surveycont. Do you want to continue taking this survey?

- Yes (1)
- I no longer wish to participate (2)

End of Block: Health & Well-Being

Start of Block: Additional Demographics

|  |
| --- |

income What is your current salary?

- Below $5,000 (1)
- $5,000 - $9,999 (2)
- $10,000 - $19,999 (3)
- $20,000 - $29,999 (4)
- $30,000 - $39,999 (5)
- $40,000 - $49,999 (6)
- $50,000 - $59,999 (7)
- $60,000 - $69,999 (8)
- $70,000 - $79,999 (9)
- $80,000 - $89,999 (10)
- $90,000 - $99,999 (11)
- $100,000 or higher (12)
- I don't know (999)
- I prefer not to say (0)

|  |
| --- |

educ What is the highest degree or level of education you have completed?

- Some high school (1)
- High school (2)
- Some college (3)
- Associate's degree (4)
- Bachelor's degree (5)
- Some graduate school (6)
- Master's degree (7)
- Ph/D/ or other Doctor program (8)
- Trade school (9)
- Other: (10) __________________________________________________
- I prefer not to respond. (999)

|  |
| --- |

livingarea What type of area do you live in?

- Remote (1)
- Rural (2)
- Suburban (3)
- Urban (4)

End of Block: Additional Demographics
