# Supplementary material for: Assessment of Heterosexual-Identified Men Who Have Sex With Men and Men of Diverse Sexual Identities: Protocol for an International, Multilingual, Online, Comparative Sexuality Study
Source: JMIR Res Protoc. 2025 Apr 30;14:e66897. doi: 10.2196/66897 (PMC12079075; doi:10.2196/66897)
Supplement: Multimedia Appendix 2 [file resprot_v14i1e66897_app2.docx]

**Multimedia Appendix 2.** Interview questionnaire.

Thank you for participating in this interview. I’m Dr. Andrew Eaton, principal investigator of this study. Your participation in today’s interview will involve discussing your sexual orientation and sexual activity. You may find some questions uncomfortable. You can skip any questions that you do not wish to answer, and you can also stop the interview at any time. I expect that the full interview will last approximately one hour. [Go over the consent form]. Do you have any questions about the consent form? Do you consent to participate in this study?

Identity Development

1. First, I have some questions regarding your sexual orientation. To begin with, how do you identify your sexual orientation?

**Probes**

- Has your sexual identity changed over time?
- Does your sexual identity change depending on context or situation?

1. Could you tell me about the process of developing your sexual orientation?

**Probes**

- What was your experience of adolescence and puberty?
- Did you sexual orientation develop in adolescence, or later in life?
- Did your family have an impact on the development of your sexual orientation in childhood or adolescence?

Attraction

1. Now I have some questions about attraction. In terms of gender, who are you usually attracted to?

**Probes**

- How has your attraction to others changed over time?
- How is your attraction to men different from your attraction to women?
- Do you feel attracted to non-binary, gender-fluid, and/or trans people? If so, how would you describe your attraction to them?

1. What emotions or feelings do you associate with your attraction to men?

Behaviour

1. This section is about your sexual behaviour. Thinking about the past 6 months, who have you had sexual encounters with?
2. In what settings do you have sexual encounters with others?

**Probes**

- Home, Sexualized Spaces, Online, Public Spaces

1. How do your sexual encounters with women differ from your sexual encounters with men?
2. Do you access any programs or services regarding sexual behaviour?

**Probes**

- STBBI Testing
- Any other community or clinical services

Relationships

1. Moving onto the topic of relationships, who are currently in an intimate or romantic relationship with?

**Probes**

- Nature of each relationship (e.g., committed, casual, co-habitating, infrequent, etc.).
- Feelings associated with each relationship

1. [If participant identifies multiple relationships] How do you navigate multiple relationships?
2. How do you communicate about sex and sexuality in your relationship(s)?

Technology Usage

1. Finally, I have some questions about technology usage. Do you use technology, such as a mobile phone, to seek sex with other men?

**Probes**

- Specific apps or websites
- Privacy strategies

1. Have you ever accessed information or support related to sex and sexuality, via technology?

**Probes**

- Types of information or support

1. What type of information regarding sex and sexuality may you want to access?

**Probes**

- Method of access
- Content of information
- Design of information

1. Would you wish to access any type of support regarding sex and sexuality, such as counselling or a peer group?

**Probes**

- Method of access
- Type of support
