# Supplementary material for: Assessment of Heterosexual-Identified Men Who Have Sex With Men and Men of Diverse Sexual Identities: Protocol for an International, Multilingual, Online, Comparative Sexuality Study
Source: JMIR Res Protoc. 2025 Apr 30;14:e66897. doi: 10.2196/66897 (PMC12079075; doi:10.2196/66897)
Supplement: Multimedia Appendix 4 [file resprot_v14i1e66897_app4.pdf]

www.sshrc-crsh.gc.ca

350 Albert Street, P.O. Box 1610 | 350 rue Albert, C.P. 1610
Ottawa ON K1P 6G4 | Ottawa ON K1P 6G4

NOTICE OF DECISION

Application Information

Applicant: Andrew Eaton

Institution: University of Regina

Funding Opportunity**: Insight Grants**

Application Number: 435-2022-0887

Stream Type: STREAM B

Title: Identity development, attraction, and behaviour of heterosexually-identified men who have sex with men

Funding Decision: **Offered**

Application Results

Your scores within committee:

Challenge (40%):	4.65/ 6
Feasibility (20%):	4.55/ 6
Capability (40%):	5.05/ 6
Total Score (weighted):	14.37/ 18

An application must receive a score of 3.0 or higher for each of the three criteria to be recommended for funding.

Rank: 11/34

Sextile category within overall competition: Second

Committee Results

Committee Name:
12B - Education and social work

<u>Stream A</u>

Applications:	13
Grants Offered:	7 (53.8%)
Grants Not Offered:	6 (46.2%)
Score of last funded application:	12.62

<u>Stream B</u>

Applications:	34
Grants Offered:	16 (47.1%)
Grants Not Offered:	18 (52.9%)
Score of last funded application:	13.68

Overall Competition Results

Applications:	1084
Grants Offered:	560 (51.7%)
Grants Not Offered:	524 (48.3%)

Sextile Categories:

First:	181 Offered / 0 Not Offered
Second:	181 Offered / 0 Not Offered
Third:	181 Offered / 0 Not Offered
Fourth:	17 Offered / 164 Not Offered
Fifth and Sixth:	0 Offered / 360 Not Offered

Committee Recommendation

The committee recommended that this meritorious proposal be funded with a budget reduction.

The applicant mentioned in their detailed description that “while this study is not solely focused on Indigenous peoples, there is a chance that some Indigenous H-MSM may participate.”

As the applicant indicated that this proposal involved Indigenous Research as defined by SSHRC, the committee would have liked to see a clearer commitment of participation of Indigenous Peoples in this project.

Questions? Email: <u>insightgrants@sshrc-crsh.gc.ca</u>

SSHRC CRSH

Committee: 12B - Education and social work
Applicant Name: Andrew Eaton
Application Number: 435-2022-0887
Assessor Number: 3

Insight Grants

External Assessment Form

Assessment

Instructions: Evaluations by external assessors are intended to assist the committee in its deliberations. SSHRC is extremely grateful for your expertise as well as your time and effort.

Given the competitive nature of the adjudication process, constructive criticism and/or suggestions for improvement, if appropriate, may be helpful to the applicant.

As your assessment will be made available to the applicant, please do not include any personal identifying information. If such information appears in your document, the Council reserves the right to remove it.

Declarations on confidentiality and conflict of interest

1) The information provided in the applications is protected by Canada's *Privacy Act* and is made available to external assessors for reviewing purposes only. I therefore agree to treat as strictly confidential all the material from the above-mentioned file which has been submitted to me by the Council. After responding, be it positively or negatively, I will ensure the destruction of the said material.

Agree X

2) I attest that I am not in a conflict of interest with the applicant(s).

Agree X

Using the guidelines in the attached document, evaluate each sub-criteria below and check the appropriate box. Briefly explain your response.

Social Sciences and Humanities Research Council of Canada
Conseil de recherches en sciences humaines du Canada
PROTECTED B WHEN COMPLETED / PROTÉGÉ B UNE FOIS REMPLI
Canada
Form/Formulaire 140

Committee: 12B - Education and social work
Applicant Name: Andrew Eaton
Application Number: 435-2022-0887
Assessor Number: 3

Part 1: Challenge — The aim and importance of the endeavour

Sub-criteria (No specific weighting assigned to each sub-criterion)	N/A	Unsatisfactory	Good	Very Good	Excellent
Originality, significance and expected contribution to knowledge					X
Appropriateness of the literature review		X			
Appropriateness of the theoretical approach or framework				X	
Appropriateness of the methods/approach					X
Quality of training and mentoring to be provided to students, emerging scholars and other highly qualified personnel, and opportunities for them to contribute					X
Potential for the project results to have influence and impact within and/or beyond the social sciences and humanities research community					X

Briefly describe the proposal's strengths and weaknesses in relation to the above criteria:

This project has the potential to substantially contribute to the literature on straight-identified men who have sex with men (MSM) and, more generally, literature about sexual minority men's wellbeing. Given public interest in the topic of straight-identified MSM, its findings have the potential to be broadly disseminated outside of higher education and highly cited within higher education. The mixed-methods approach is appropriate and feasible, as is the plan for graduate student training. The plan to interview straight-identified MSM and disseminate a survey will harness the strengths of both qualitative and quantitative research. This project will substantially contribute to knowledge, and I highly support it.

I do have some concerns about the project related to the applicants' grasp of the relevant literature on this topic. I will divide these comments into separate sections.

(1) The applicants have not incorporated several key studies about straight-identified MSM, and as a result, they are unable to use the insights from those projects to shape their own. For instance, the proposal makes two incorrect statements: (1) "Past research in other countries with self-identified H-MSM participants has utilized small samples (i.e., 8-10 participants) or is more than a decade old" and (2) "While the potential reasons for discord between sexual identity and behaviour have not yet been fulsomely explored amongst H-MSM…"

In fact, extensive qualitative and quantitative research has been conducted about straight-identified MSM, focusing on topics as varied as why they identify as straight and the relationship between health outcomes and discordance. Without using this prior research as a foundation for their project, the applicants risk asking questions that have already been addressed. Of course, the authors also propose an entirely new setting (Canada) and important measures not previously explored. Nonetheless, there is some ground other researchers have covered, and the applicants need to incorporate those insights to shape their project so that it can reach its full potential. See for instance:

Committee: 12B - Education and social work
Applicant Name: Andrew Eaton
Application Number: 435-2022-0887
Assessor Number: 3

Caplan, Zoe. 2017. “The Problem with Square Pegs: Sexual Orientation Concordance as a Predictor of Depressive Symptoms.” Society and Mental Health 7 (2): 105–20. https://doi.org/10.1177/2156869317701266.

Carrillo, Héctor, and Amanda Hoffman. 2016. “From MSM to Heteroflexibilities: Non-Exclusive Straight Male Identities and Their Implications for HIV Prevention and Health Promotion.” Global Public Health 11 (7–8): 923–36. https://doi.org/10.1080/17441692.2015.1134272.

———. 2018. “‘Straight with a Pinch of Bi’: The Construction of Heterosexuality as an Elastic Category among Adult US Men.” Sexualities 21 (1–2): 90–108. https://doi.org/10.1177/1363460716678561.

Cerna, Jonathan de la, and Karl Jade Cosido. 2020. “His Other Man: Straight Men’s Romantic Relationships with Homosexual Partners.” Asia Pacific Journal of Academic Research in Social Sciences 5 (1): 21–34.

Fu, Tsung-chieh, Debby Herbenick, Brian Dodge, Christopher Owens, Stephanie A. Sanders, Michael Reece, and J. Dennis Fortenberry. 2019. “Relationships Among Sexual Identity, Sexual Attraction, and Sexual Behavior: Results from a Nationally Representative Probability Sample of Adults in the United States.” Archives of Sexual Behavior 48 (5): 1483–93. https://doi.org/10.1007/s10508-018-1319-z.

Gattis, Maurice N., Paul Sacco, and Renee M. Cunningham-Williams. 2012. “Substance Use and Mental Health Disorders Among Heterosexual Identified Men and Women Who Have Same-Sex Partners or Same-Sex Attraction: Results from the National Epidemiological Survey on Alcohol and Related Conditions.” Archives of Sexual Behavior 41 (5): 1185–97. http://dx.doi.org/10.1007/s10508-012-9910-1.

Geary, Rebecca S., Clare Tanton, Bob Erens, Soazig Clifton, Philip Prah, Kaye Wellings, Kirstin R. Mitchell, et al. 2018. “Sexual Identity, Attraction and Behaviour in Britain: The Implications of Using Different Dimensions of Sexual Orientation to Estimate the Size of Sexual Minority Populations and Inform Public Health Interventions.” PLOS ONE 13 (1): e0189607. https://doi.org/10.1371/journal.pone.0189607.

Krueger, Evan A., and Dawn M. Upchurch. 2019. “Are Sociodemographic, Lifestyle, and Psychosocial Characteristics Associated with Sexual Orientation Group Differences in Mental Health Disparities? Results from a National Population-Based Study.” Social Psychiatry and Psychiatric Epidemiology 54 (6): 755–70. https://doi.org/10.1007/s00127-018-1649-0.

Kuperberg, Arielle, and Alicia M. Walker. 2018. “Heterosexual College Students Who Hookup with Same-Sex Partners.” Archives of Sexual Behavior 47 (5): 1387–1403. https://doi.org/10.1007/s10508-018-1194-7.

Lourie, Michael A., and Belinda L. Needham. 2017. “Sexual Orientation Discordance and Young Adult Mental Health.” Journal of Youth and Adolescence 46 (5): 943–54. https://doi.org/10.1007/s10964-016-0553-8.

Mendelsohn, David M., Allen M. Omoto, Karen Tannenbaum, and Christopher S. Lamb. 2021. “When Sexual Identity and Sexual Behaviors Do Not Align: The Prevalence of Discordance and Its Physical and Psychological Health Correlates.” Stigma and Health. https://doi.org/10.1037/sah0000338.

Mishel, Emma. 2019. “Intersections between Sexual Identity, Sexual Attraction, and Sexual Behavior among a Nationally Representative Sample of American Men and Women.” Journal of Official Statistics 35 (4): 859–84. https://doi.org/10.2478/jos-2019-0036.

Richters, Juliet, Dennis Altman, Paul B. Badcock, Anthony M. A. Smith, Richard O. de Visser, Andrew E. Grulich, Chris Rissel, and Judy M. Simpson. 2014. “Sexual Identity, Sexual Attraction and Sexual Experience: The Second Australian Study of Health and Relationships.” Sexual Health 11 (5): 451–60. https://doi.org/10.1071/SH14117.

Silva, Tony. 2021. Still Straight: Sexual Flexibility among White Men in Rural America. New York: NYU Press.

———. 2019. “Straight Identity and Same-Sex Desire: Conservatism, Homophobia, and Straight Culture.” Social Forces 97 (3): 1067–94. https://doi.org/10.1093/sf/soy064.

(2) The authors explain that hegemonic masculinity theory is a key framework they will use in their analysis. This framework is appropriate, but I recommend that the authors more fully engage with hegemonic masculinity theory, because it seems like they equate hegemonic masculinity to “stereotypically male traits,” when in fact the concept is more complicated. See for instance:

Messerschmidt, James W. 2019. “The Salience of ‘Hegemonic Masculinity.’” Men and Masculinities 22 (1): 85–91. https://doi.org/10.1177/1097184X18805555.

(3) I noticed some misuse of technical language. Perhaps these misuses were accidental, but the applicants need to be careful to ensure that they explain the project correctly—both to academic audiences and to participants. For instance, the authors refer to “the anonymous and confidential nature of our research,” when in fact anonymous and confidential mean two completely different things. Such misunderstandings could mislead participants. Do the applicants mean that some options for participation will be entirely anonymous (e.g., in an anonymous online chatroom or over the phone with the participant’s number blocked, their voice distorted, and the avoidance of questions that could expose sensitive information) whereas other options will be confidential (i.e., kept secret by researchers)? Please explain.

The applicants also note that “Following the qualitative phase, we will conduct an online quantitative survey with a target 250 H-MSM geographically dispersed across Canada to better understand this population with a representative sample.” No survey sample is representative unless researchers employ probability sampling of the target population.

(4) The applicants propose to measure sexuality using the Kinsey Scale, when the field has moved beyond that measure. It is important to separately measure attraction, behaviour, and identity with well-validated measures. See for instance questions about sexual attraction from the National Survey of Drug Use and Health (NSDUH) or the proposed measures from Statistics Canada (https://www.statcan.gc.ca/en/concepts/consult-variables/gender).

Part 2: Feasibility — The plan to achieve excellence

Committee: 12B - Education and social work
Applicant Name: Andrew Eaton
Application Number: 435-2022-0887
Assessor Number: 3

Sub-criteria (No specific weighting assigned to each sub-criterion)	N/A	Unsatisfactory	Good	Very Good	Excellent
Appropriateness of the proposed timeline, and probability that the objectives will be met					X
Expertise of the applicant or team in relation to the proposed research					X
Appropriateness of the requested budget, justification of proposed costs, and, where applicable, other cash and/or in-kind contributions		X			
Quality and appropriateness of knowledge mobilization plans, including effective dissemination, exchange and engagement with stakeholders within and/or beyond the research community, where applicable					X

Briefly describe the proposal's strengths and weaknesses in relation to the above criteria:

The project is highly feasible within the specified timeframe. The plans for knowledge mobilization are appropriate. The team is composed of experts in a variety of subfields, which will guarantee that the project will be able to accomplish its goals. The applicants are productive, ambitious experts who have made a large impact on their fields and will continue to do so.

I do have one concern about the budget, and a big one. I am not convinced that a project coordinator is necessary. The expense of the project coordinator is enormous relative to other expenses, and I see no clear rationale for this expense given that there are two applicants (one primary) and several collaborators. Among them there is ample expertise and time to coordinate this project. Related expenses (e.g., a computer for the coordinator) are also unnecessary. To put the project into perspective, its data goals (40 qualitative interviews and 250 survey participants) is about in line with a dissertation project—which does not require a coordinator, particularly given the team of highly successful experts on board.

Part 3: Capability — The expertise to succeed

In the case of a research team, evaluate each team member's research achievements **(do not include collaborators).**

In your evaluation, address the following criteria while **considering the applicant's and/or team members' stage of career**:

Sub-criteria (No specific weight assigned to each sub-criterion)	N/A	Unsatisfactory	Good	Very Good	Excellent

Committee: 12B - Education and social work
Applicant Name: Andrew Eaton
Application Number: 435-2022-0887
Assessor Number: 3

Sub-criteria (No specific weight assigned to each sub-criterion)	N/A	Unsatisfactory	Good	Very Good	Excellent
Quality, quantity and significance of past experience and published and/or creative outputs of the applicant and any co-applicants, relative to their roles in the project and to the stage of their career					X
Evidence of past knowledge mobilization activities (e.g. films, performances, commissioned reports, knowledge syntheses, experience in collaboration/other interactions with stakeholders, contributions to public debate and media), and of impacts on professional practice, social services and policies, etc.					X
Quality and quantity of past contributions to the development of training and mentoring of students, postdoctoral researchers and other highly qualified personnel					X

Briefly describe the proposal's strengths and weaknesses in relation to the above criteria:

Dr. Eaton is a highly accomplished scholar with an impressive record of publications, grants, and conference participation. Dr. Eaton also began to actively train graduate students when he started his new position only months ago. In short, Dr. Eaton is highly accomplished and clearly able to successfully pursue this project. Dr. Shuper is also highly accomplished, with a record of impressive accomplishments including important research positions, grants, and publications. These two will make an excellent team who will successfully pursue this project with collaborators.

Part 4: Additional Comments

If you have comments regarding the budget or other aspects of the proposal, please include them here:

SSHRC CRSH

**Committee:**	12B - Education and social work
**Applicant Name:**	Andrew Eaton
**Application Number:**	435-2022-0887
**Assessor Number:**	6

Insight Grants

External Assessment Form

Assessment

Instructions: Evaluations by external assessors are intended to assist the committee in its deliberations. SSHRC is extremely grateful for your expertise as well as your time and effort.

Given the competitive nature of the adjudication process, constructive criticism and/or suggestions for improvement, if appropriate, may be helpful to the applicant.

As your assessment will be made available to the applicant, please do not include any personal identifying information. If such information appears in your document, the Council reserves the right to remove it.

Declarations on confidentiality and conflict of interest

1) The information provided in the applications is protected by Canada's *Privacy Act* and is made available to external assessors for reviewing purposes only. I therefore agree to treat as strictly confidential all the material from the above-mentioned file which has been submitted to me by the Council. After responding, be it positively or negatively, I will ensure the destruction of the said material.

Agree X

2) I attest that I am not in a conflict of interest with the applicant(s).

Agree X

Using the guidelines in the attached document, evaluate each sub-criteria below and check the appropriate box. Briefly explain your response.

Social Sciences and Humanities Research Council of Canada
Conseil de recherches en sciences humaines du Canada
PROTECTED B WHEN COMPLETED / PROTÉGÉ B UNE FOIS REMPLI
Canada
Form/Formulaire 140

Committee: 12B - Education and social work
Applicant Name: Andrew Eaton
Application Number: 435-2022-0887
Assessor Number: 6

Part 1: Challenge — The aim and importance of the endeavour

Sub-criteria (No specific weighting assigned to each sub-criterion)	N/A	Unsatisfactory	Good	Very Good	Excellent
Originality, significance and expected contribution to knowledge					X
Appropriateness of the literature review					X
Appropriateness of the theoretical approach or framework					X
Appropriateness of the methods/approach					X
Quality of training and mentoring to be provided to students, emerging scholars and other highly qualified personnel, and opportunities for them to contribute					X
Potential for the project results to have influence and impact within and/or beyond the social sciences and humanities research community					X

Briefly describe the proposal's strengths and weaknesses in relation to the above criteria:

Research on H-MSM is greatly needed, as such the significance of the potential contributions is high. The applicants have a strong grasp of the limited literature on this group of men. The use of hegemonic masculinity theory and minority stress theory are quite appropriate and their integration is important for this study group. I find their plan to use sequential mixed methods to be very appropriate. They will first examine the topic phenomenologically through a carefull analysis of qualitative interviews is important. Starting with qualitative data can enable the team to modify elements of the second stage, the survey that will yield quantitative data. The analysis of both qualitative and quantitative data will offer a more comprehensive understanding of this group of men than either method alone. As a graduate student I was involved in multi-year grant funded research and can attest the importance of this experience for my career and teaching. The graduate students will learn from hands on experience as to the research process, they will be able to use their increased knowledge in their own research, teaching, or community/policy work in the future. The research questions being asked in this proposal are very important and will certainly contribute to the relatively small literature on this topic. Our understanding of men who hold one identity but behave in a manner inconsistent with that identity is important for theory, practice, and policy. I suspect that there are other types of identities and behaviors that are incongruent in people and this research may even promote more research on this pattern for other topics not just H-MSM. I see no weakness in the proposal regarding the above criteria.

Part 2: Feasibility — The plan to achieve excellence

Sub-criteria (No specific weighting assigned to each sub-criterion)	N/A	Unsatisfactory	Good	Very Good	Excellent

Committee: 12B - Education and social work
Applicant Name: Andrew Eaton
Application Number: 435-2022-0887
Assessor Number: 6

Sub-criteria (No specific weighting assigned to each sub-criterion)	N/A	Unsatisfactory	Good	Very Good	Excellent
Appropriateness of the proposed timeline, and probability that the objectives will be met					X
Expertise of the applicant or team in relation to the proposed research					X
Appropriateness of the requested budget, justification of proposed costs, and, where applicable, other cash and/or in-kind contributions					X
Quality and appropriateness of knowledge mobilization plans, including effective dissemination, exchange and engagement with stakeholders within and/or beyond the research community, where applicable					X

Briefly describe the proposal's strengths and weaknesses in relation to the above criteria:

The timeline seems appropriate to me and well though out. The team is interdisciplinary and each individual brings something of great value to the team. As a whole, the team is highly experienced in research on this and related topics and on the proposed methods. The PI has the experience and publication record to manage the team and time line. The team has publications and experience is using the methods of recruitment, interviews, and surveys that are proposed. The requested budget is very appropriate and I am impressed that there are several offers of in-kind funding from the individual's organziations/universities. The team has proposed an excellent 5 point knowledge mobilization plan with realistic goals and expectations for dissemination and exchange and engagement with stakeholders.

Part 3: Capability — The expertise to succeed

In the case of a research team, evaluate each team member's research achievements **(do not include collaborators).**

In your evaluation, address the following criteria while **considering the applicant's and/or team members' stage of career**:

Sub-criteria (No specific weight assigned to each sub-criterion)	N/A	Unsatisfactory	Good	Very Good	Excellent
Quality, quantity and significance of past experience and published and/or creative outputs of the applicant and any co-applicants, relative to their roles in the project and to the stage of their career					X

Committee: 12B - Education and social work
Applicant Name: Andrew Eaton
Application Number: 435-2022-0887
Assessor Number: 6

Sub-criteria (No specific weight assigned to each sub-criterion)	N/A	Unsatisfactory	Good	Very Good	Excellent
Evidence of past knowledge mobilization activities (e.g. films, performances, commissioned reports, knowledge syntheses, experience in collaboration/other interactions with stakeholders, contributions to public debate and media), and of impacts on professional practice, social services and policies, etc.					X
Quality and quantity of past contributions to the development of training and mentoring of students, postdoctoral researchers and other highly qualified personnel					X

Briefly describe the proposal's strengths and weaknesses in relation to the above criteria:

The team has the expertise to succeed. In brief, the PI is expectionally prepared to train and mentor graduate students having had the opportunity as a doctoral student to supervise numerous research assistants and supervising a research lab. The PI has a publication record of a late career scholar despite starting their first academic appointment in 2021; this is quite exceptional. The relevance of their publications is manifestly clear for example the PI has a publication on training researchers for community-based participatory research - a training that has been implemented in 5 other locations, and a publication on youths use of technology which reveals expertise in understanding how the current subject pool may use technology in the form of on-line communities. Fianlly, the PI has also developed at least one intervention and successfully published the research supporting it which led to the development of a new staff person to oversee and implement the intervention. The Co-I is also expectionally well suited to execute this project. They are highly published and highly regarded internationally, have been asked to develop interventions and has worked with the WHO to develop policy and interventions. The Co-I is also experienced in the 5 points of the KM plan showing tremendous experience with engaging and communicating with stakeholders. In addition to developing several interventions, this peson has also engaged in community-based research, skills which will allow the team to succeed.

Part 4: Additional Comments

If you have comments regarding the budget or other aspects of the proposal, please include them here:

I think this is an extremely well developed proposal, with an appropriate time-line and budget. The team is excellently suited to reach excellence and success. The project is timely as scholars and practicioners are increasingly aware of this group of men (H-MSM) but not enough is known about the development of their identities when their behavior seems incongruent and little is known about the psychological, social, economic, and health consequences of this disconjunction.
